# PDGFRβ^+^ cells play a dual role as hematopoietic precursors and niche cells during mouse ontogeny

**DOI:** 10.1016/j.celrep.2022.111114

**Published:** 2022-07-19

**Authors:** Diana Sá da Bandeira, Alastair Morris Kilpatrick, Madalena Marques, Mario Gomez-Salazar, Telma Ventura, Zaniah Nashira Gonzalez, Dorota Stefancova, Fiona Rossi, Matthieu Vermeren, Chris Sebastiaan Vink, Mariana Beltran, Neil Cowan Henderson, Bongnam Jung, Reinier van der Linden, Harmen Jan George van de Werken, Wilfred F.J. van Ijcken, Christer Betsholtz, Stuart John Forbes, Henar Cuervo, Mihaela Crisan

**Affiliations:** 1 Centre for Cardiovascular Science, The Queen’s Medical Research Institute, University of Edinburgh, EH16 4TJ Edinburgh, UK; 2 Centre for Regenerative Medicine, Institute for Regeneration and Repair, University of Edinburgh, 5 Little France Drive, EH16 4UU Edinburgh, UK; 3 Centre for Inflammation Research, Institute for Regeneration and Repair, The Queen’s Medical Research Institute, University of Edinburgh, EH16 4TJ Edinburgh, UK; 4 MRC Human Genetics Unit, Institute of Genetics and Molecular Medicine, University of Edinburgh, EH4 2XU Edinburgh, UK; 5 Department of Immunology, Genetics, and Pathology, Uppsala University, 751 85 Uppsala, Sweden; 6 Harvard Medical School, Department of Surgery, Boston Children’s Hospital, Boston, MA 02115, USA; 7 Hubrecht Institute, Department van Oudenaarden Quantitative Biology, 3584 Utrecht, the Netherlands; 8 Erasmus MC Cancer Institute, University Medical Center, Cancer Computational Biology Center, and Departments of Urology and Immunology, 3000 Rotterdam, the Netherlands; 9 Center for Biomics, Department of Cell Biology, Erasmus MC University Medical Centre, 3015 Rotterdam, the Netherlands; 10 Department of Medicine Huddinge, Karolinska Institutet, 141 57 Huddinge, Sweden; 11 Department of Physiology and Biophysics, College of Medicine, University of Illinois at Chicago, Chicago, IL 60612, USA

**Keywords:** PDGFRβ, pericytes, VSMCs, hematopoietic niche, MSCs, osteogenesis, HSPC precursor, Bmp4, AGM single-cell RNA-sequencing

## Abstract

Hematopoietic stem cell (HSC) generation in the aorta-gonad-mesonephros region requires HSC specification signals from the surrounding microenvironment. In zebrafish, PDGF-B/PDGFRβ signaling controls hematopoietic stem/progenitor cell (HSPC) generation and is required in the HSC specification niche. Little is known about murine HSPC specification *in vivo* and whether PDGF-B/PDGFRβ is involved. Here, we show that PDGFRβ is expressed in distinct perivascular stromal cell layers surrounding the mid-gestation dorsal aorta, and its deletion impairs hematopoiesis. We demonstrate that PDGFRβ^+^ cells play a dual role in murine hematopoiesis. They act in the aortic niche to support HSPCs, and in addition, PDGFRβ^+^ embryonic precursors give rise to a subset of HSPCs that persist into adulthood. These findings provide crucial information for the controlled production of HSPCs *in vitro*.

## Highlights

• PDGFRβ deletion affects hematopoietic development in the AGM *in vivo*
• The transcriptome and hematopoietic support of the PDGFRβ-KO niche are altered
• The osteogenic gene profile and differentiation of KO AGM MSCs are affected
• PDGFRβ^+^ early embryonic precursors contribute to EC and HSPC lineages *in vivo*

## Graphical abstract

**Figure:**
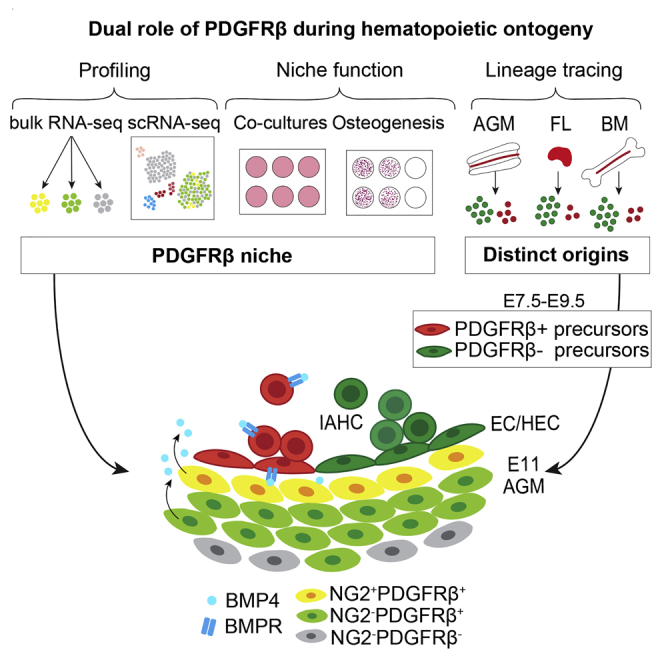

## Teaser

Sá da Bandeira et al. find that PDGFRβ, expressed in the AGM hematopoietic microenvironment, controls HSPCs. By RNA sequencing, hematopoietic assays, and lineage tracing, they show that PDGFRβ has a dual role: it is required in the AGM hematopoietic niche and marks early precursors of a subset of ECs and HSPCs.

## Introduction

Hematopoietic stem cells (HSCs) are indispensable for life. They are first detected in the aorta-gonad-mesonephros (AGM) region of the mouse embryo, at embryonic day (E)10.5 (Boisset et al., 2010; Chen et al., 2009; Costa et al., 2012; Crisan and Dzierzak, 2016; de Bruijn et al., 2002; Jaffredo et al., 1998; Medvinsky and Dzierzak, 1996; Oberlin et al., 2002; Tavian et al., 2001; Zovein et al., 2008) These cells arise from hemogenic endothelial cells (HECs) that undergo an endothelial-to-hematopoietic transition (EHT). Shortly after, HSCs migrate to the fetal liver (FL), where they expand and mature before permanently migrating to the bone marrow (BM) from E15.5 on (Ciriza et al., 2013). Other intra- and extra-embryonic locations temporarily contain hematopoietic stem and progenitor cells (HSPCs), such as the head (Li et al., 2012, 2016), yolk sac (YS), and placenta (PL) (Gekas et al., 2010; Ottersbach and Dzierzak, 2005).

Signals from the microenvironment are required for HSPC generation. *In vitro* studies have shown that stromal cell lines derived from E11 AGMs support hematopoiesis in co-cultures with BM HSPCs (Oostendorp et al., 2002a, 2002b). This was further confirmed *in vivo* in 2013, when Richard et al. (2013) demonstrated that the interaction between the subaortic mesenchyme and the endothelium in the chick embryo was necessary to induce Runx1 expression, a transcription factor required for HSPC generation (Chen et al., 2009). Altogether, these studies suggest that mesenchymal stromal cells play a role in the emergence of HSPCs. However, the nature of the cells and signals involved remain poorly understood.

The HSPC microenvironment comprises pericytes/vascular smooth muscle cells (PCs/VSMCs) and other cells. PCs are known to support HSPCs in the adult BM (Corselli et al., 2013; Ding et al., 2012; Sá da Bandeira et al., 2016). For example, Sacchetti et al. (2007) demonstrated that CD146^+^ perivascular cells express *Jagged1*, *N-Cadherin*, *Cxcl12*, and *Scf* molecules, implicated in adult HSPC maintenance. Moreover, Corselli et al. (2013) showed that CD146^+^ but not CD146^−^ cells support transplantable human hematopoietic stem cell potential in culture. In the mouse BM, pericytes expressing nestin, neural/glial antigen 2 (NG2), or leptin receptor were found to support HSPCs and express supportive niche genes such as *Cxcl12*, *Adrb3*, *Scf*, *Angtp1*, *Vcam1*, *and Opn* (Ding et al., 2012; Kunisaki et al., 2013; Mendez-Ferrer et al., 2010; Pinho et al., 2013).

Whether perivascular cells are also involved in embryonic hematopoietic development *in vivo* is unknown. Pericytes are recruited to the vascular wall via platelet-derived growth factor (PDGF)-B/PDGF receptor (PDGFR)β signaling (Hellstrom et al., 1999, 2001; Lindahl et al., 1997) and microarray screening of HSPC-supportive AGM-derived stromal cell lines showed that both *Mcam* (CD146) and *Pdgfrb* were expressed in these cells (Charbord et al., 2014). *In vivo*, PDGFRβ and CD146 are expressed by perivascular cells in close contact with the mid-gestation dorsal aorta (DA) (Azzoni et al., 2018; Roostalu et al., 2018). However, their role in hematopoietic cell development in the AGM was never investigated. Moreover, in zebrafish, PDGF signaling contributes to the HSC specification niche (Damm and Clements, 2017) by mediating the migration of neural crest cells to the DA, which in turn, specify HSCs. In addition, morpholino knockdown of *pdgfrb* or dominant-negative *pdgfrb* expression reduced AGM HSPCs (Lim et al., 2017).

We thus hypothesized that PDGFRβ^+^ perivascular cells control hematopoietic development in the mouse AGM. To test this, we characterized the perivascular cells surrounding the mid-gestation DA, tested their niche activity, and traced them throughout hematopoietic ontogeny. Our results demonstrate that PDGFRβ^+^ AGM cells support HSPC activity, and early embryonic cells contribute to both endothelial and hematopoietic cell lineages during ontogeny.

## Results

### The embryonic DA is surrounded by phenotypically and transcriptomically distinct perivascular cell layers

**Figure 1:**
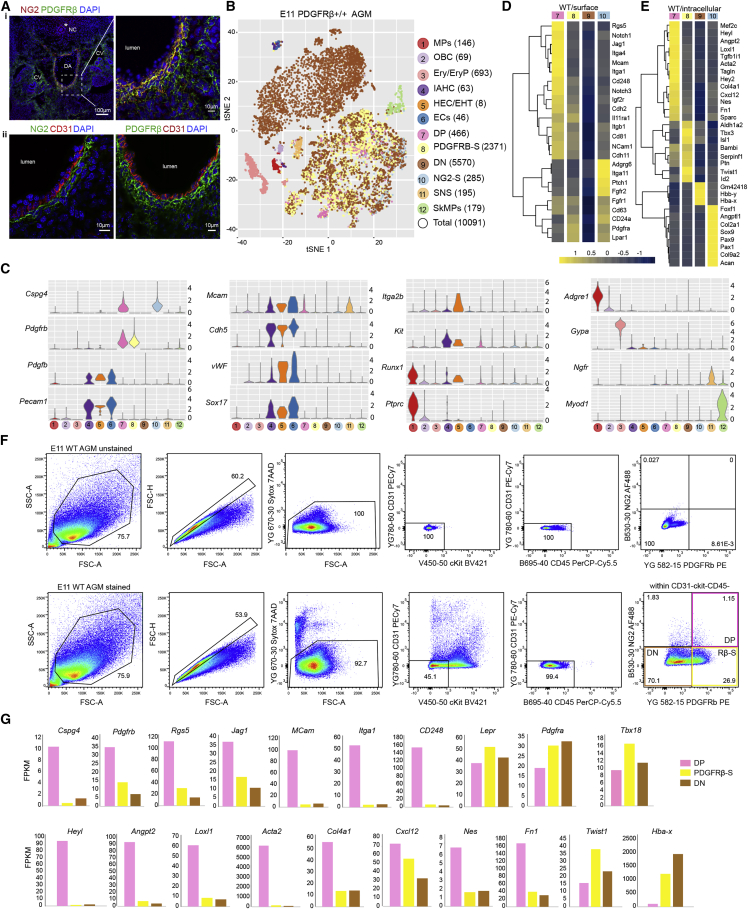
Distinct phenotypic and transcriptomic perivascular cell subsets surround the midgestation DA (A) Immunohistochemistry of E11 WT DA, showing NG2 (i, ii), PDGFRβ (i, ii), and CD31 (ii) expression. Nuclei were counterstained with DAPI. CV, cardinal veins; NC, notochord. (B) t-distributed stochastic neighbor embedding (t-SNE) plots showing 12 E11 WT cell populations and their numbers (42sp). (C) Violin plots showing the expression of genes used to identify the cell clusters DP, PDGFRβ-S, DN, and NG2-S. MP, macrophages; OBC, other blood cells; Ery/EryP, erythroid/progenitors; IAHC, intra-aortic hematopoietic clusters; HEC/EHT, hemogenic endothelial cells/endothelial-to-hematopoietic transition; EC, endothelial cells; SNS, sympathetic nervous system; SkMP, skeletal muscle progenitors. (D and E) Heatmaps showing gene expression of differentially expressed genes encoding surface (D) and intra-/extracellular (E) proteins enriched in each of the NG2^+/−^PDGFRβ^+/−^ populations. (F) Isolation of perivascular cells. (G) Fragments per kilobase of transcript per million mapped reads (FPKM) values of selected genes by bulk RNA-seq.

We characterized the AGM microenvironment by immunohistochemistry using a combination of known endothelial and perivascular cell markers. We found that the DA is surrounded by a layer of perivascular cells co-expressing NG2, a proteoglycan expressed by pericytes during vascular morphogenesis (Ozerdem et al., 2001), and PDGFRβ, a receptor involved in pericyte recruitment and blood vessel stabilization (Armulik et al., 2005) and expressed in perivascular cells in the AGM (Azzoni et al., 2018). These PCs/VSMCs, hereafter referred to as double-positive (DP, yellow, Figure 1Ai), do not express CD31, a marker for ECs and the intra-aortic hematopoietic cluster (IAHC) (Figure 1Aii). Surrounding DP cells, we detected PDGFRβ^+^NG2^−^ perivascular cells that we called PDGFRβ-single (PDGFRβ-S, green, Figure 1Ai), and more distally, other stromal cells low/negative for these markers, which we called double-negative (DN) (Figure 1Ai). Cells expressing only NG2 were detected around the notochord (star), mainly in the trunk, and were here called NG2-single (NG2-S) (Figure 1Ai).

To better characterize these perivascular cells that compose the HSPC-generating microenvironment, we performed single-cell RNA sequencing (scRNA-seq) analysis of whole E11 AGMs. We used graph-based clustering and known marker expression and identified 12 major populations in the E11 wild-type (WT) AGM (Figure 1B). We confirmed the expression of *Pdgfrb* and *Cspg4* (NG2) in the 4 populations we identified by immunohistochemistry that include DP (7), PDGFRβ-S (8), DN (9), and NG2-S cells (10) (Figure 1C). The cells associated with IAHCs (cluster 4) were identified as *Pecam1*(CD31)^*+*^*Kit*^+^, as previously shown (Solaimani Kartalaei et al., 2015; Yokomizo and Dzierzak, 2010). Runx1 expression, seen in HECs, including those entering EHT (Chen et al., 2009; Gao et al., 2018), allowed us to separate cluster 5 enriched in these cells (HEC/EHT, *Pecam1*^*+*^*Ptprc*(CD45)^−^*Kit*^*low/*−^*Runx1*^*+*^) from the EC cluster 6 (*Pecam1*^*+*^*Ptprc*^−^*Kit*^*low/*−^*Runx1*^−^). The expression of other genes such as *Cdh5* (vascular endothelial [VE] cadherin), *vWF*, and *Sox17* further confirmed the cellular identity of hematopoietic cells and/or hemogenic cells/ECs. *Adgre1* (F4/80)^+^ macrophages (MPs), *Gypa* (CD235)^+^ erythroid and erythroid progenitors (Ery/EryP), *Ngfr*^*+*^cells found in the sympathetic nervous system (SNS) and *MyoD1*^*+*^ skeletal muscle progenitors (SkMP) were enriched in clusters 1, 3, 11, and 12, respectively (Figure 1C). The expression of *Mcam* (CD146) was confirmed in a subset of DP cells (Figure 1C). However, *Mcam* was mainly expressed by ECs (cluster 6), confirming previous work (McGarvey et al., 2017), as well as by a few cells in clusters 4, 5, and 11 (Figure 1C). A previous report showed by immunohistochemistry that ckit is expressed in a subset of mesenchymal cells around the DA (Fitch et al., 2020). Our datasets show that in addition to IAHCs (cluster 4), *Kit* is also expressed in a subset of NG2^+/−^PDGFRβ^+/−^ cells (Figure 1C, clusters 7–10).

To further characterize the different perivascular cell subsets, we compared the gene expression profiles of NG2^+/−^PDGFRβ^+/−^ cell populations. Heatmaps showed distinct expression patterns for each population (Figures 1D and 1E). Genes including *Rgs5*, *Jag1*, *Mcam*, *CD248*, *Heyl*, and *Acta2* were enriched in the DP cell population, while *Tbx3*, *Isl1*, *Twist1*, and *Id2*, transcription factors involved in heart development or angiogenesis (Moretti et al., 2006; Perkhofer et al., 2016; Washkowitz et al., 2012), were enriched in PDGFRβ-S cells. We found that *Sox9* was highly expressed in the NG2-S cell population (Figure 1E). This transcription factor, predominantly expressed in mesenchymal condensations during cartilage deposition in the mouse embryo, was associated with numerous developmental processes, including neural crest and sclerotome development (Barrionuevo et al., 2006; Cheung and Briscoe, 2003; Wright et al., 1995). Other cartilage development genes such as *Pax1*, *Pax9*, *Col2a1*, and *Acan* (Takimoto et al., 2019) were also enriched in the NG2-S population (Figure 1E), suggesting that at least some of these cells are cartilage progenitors and/or regulate chondrogenesis.

To validate whether the cell surface marker combinations we identified by immunohistochemistry and scRNA-seq (Figures 1A–E) can be used to purify perivascular cell subsets, we performed flow cytometry analysis and cell sorting. Upon negative selection of E11 AGM CD31, ckit, and CD45 cells (Figure 1F), subpopulations of NG2^+^PDGFRβ^+^ cells (DP, 1.21%), NG2^−^PDGFRβ^+^ cells (PDGFRβ-S, 28.66%), and NG2^−^PDGFRβ^low/−^ cells (DN, 68.67%) were purified and used for RNA-seq. We confirmed the presence of *Pdgfrb* (PDGFRβ), *Cspg4* (NG2), and other differentially expressed genes (DEGs) in the sorted populations (Figure 1G). Other markers such as leptin receptor (*Lepr*) and PDGFRα (*Pdgfrα*), were expressed in all purified cell subsets at various levels (Figure 1G), as was the expression of *Tbx18* (Figure 1G), a gene associated with pericytes in adult organs (Guimaraes-Camboa et al., 2017). Altogether, we were able to successfully isolate distinct perivascular cell populations that compose the E11 WT AGM based on cell surface marker combinations we identified.

### AGM HSPC activity is impaired in the absence of PDGFRβ

**Figure 2:**
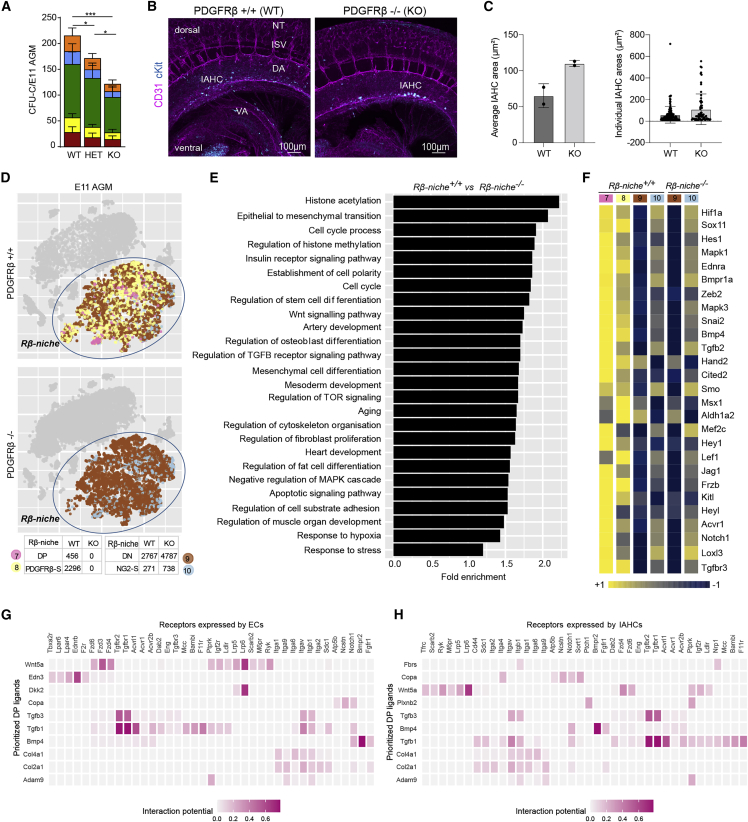
PDGFRβ deletion impairs AGM HSPC number and alters the genetic program of the niche (A) CFU-C numbers per E11 AGM; error bars: SD; ^∗^p < 0.05; ^∗∗∗^p < 0.001 (see Table S1). (B) Whole-mount immunostaining of WT/KO E10.5 AGMs (n = 2/2 embryos, N = 2 experiments) stained with CD31 and cKit. (C) The average of IAHC area (left) and individual areas of all IAHC combined (right) are shown (n = 2/2, N = 2); error bars: SD. (D) t-SNE plots showing the *Rβ-niche* cluster used for WT/KO comparisons containing clusters 7, 8, 10, and a subset of 9. (E) Selected gene ontology (GO) biological processes significantly overrepresented in genes significantly downregulated in the KO *Rβ-niche*. (F–H) Heatmap of top genes associated with the mesenchymal cell differentiation GO: 0048762 significantly downregulated in the KO *Rβ-niche* (see Table S4). Ligand-receptor interactions inferred by NicheNet between DP cells and ECs (G) or IAHCs (H). Genes from the GO term “mesenchymal cell differentiation” (E) were used as a DP gene set of interest for (G) and (H).

Because PDGFRβ is expressed by perivascular cells surrounding the aortic endothelium (Figure 1Aii), and recent evidence implicates it in zebrafish HSC ontogeny (Lim et al., 2017; Damm and Clements, 2017), we sought to determine its requirement in HSPC generation in the mouse embryo. We used PDGFRβ knockout (KO) mice, which lack pericytes and die perinatally (Soriano, 1994). In these mice, blood vessels form normally until E16, which allows us to investigate the role of PDGFRβ in early embryonic hematopoiesis in the absence of any vascular defect. We confirmed the absence of PDGFRβ expression in E10 and E11 KO AGMs (Figures S1A–S1C). To test whether PDGFRβ signaling is involved in AGM hematopoietic development, we performed hematopoietic progenitor assays using E11 AGMs. We found a significant decrease in all colony-forming unit-culture (CFU-C) types in both heterozygous (HET) and homozygous (KO) mutants compared with WT littermates (Figure 2A; Table S1), implicating PDGFRβ signaling in hematopoietic progenitor development.

To test whether HSCs were affected by the loss of PDGFRβ, we harvested AGMs from E11 PDGFRβ WT and mutant embryos and transplanted them into sublethally irradiated recipients. Of 15 recipient mice, 3 injected with WT AGMs reconstituted, whereas mice injected with HET or KO mutant AGM cells showed low (2/17) or no long-term reconstitution (0/7) (Figures S1D and S1E), suggesting the involvement of PDGFRβ in AGM HSC development.

Since PDGFRβ is expressed in other hematopoietic sites between E8 and E11 (Figures S1F and S1G), we tested whether the decline in AGM HSPCs in PDGFRβ-KO mice was aligned with deficiencies of HSPCs in other hematopoietic organs. We found no significant differences in CFU-Cs in E10 (Figure S1H; Table S2), and E11 (Figure S1I; Table S3) heads, FL, YS, and PL. However, there was a significant decrease in CFU-G/E11 KO head and blast-forming unit erythroid progenitor (BFU-E)/E11 KO YS compared to the WT littermates. Interestingly, CFU-C numbers were not affected in the E10 AGM before the onset of HSC generation (Figure S1H; Table S2), suggesting that PDGFRβ absence affects only the definitive wave of hematopoiesis. This is supported by the decrease in E11 but not E10 BFU-E/KO YS (Tables S2 and S3).

To confirm that this decrease is not due to defects in the vasculature, we performed whole-mount immunostaining of PDGFRβ WT and KO mouse embryos using both endothelial and hematopoietic cell markers (Figure 2B). The KO DA appears normal. IAHCs were also present in KO embryos, but they appear to be larger and more ventral. To assess whether HEC/EHT cells were affected in PDGFRβ-KO embryos, we crossed PDGFRβ^+/−^Runx1^GFP/GFP^ mice and analyzed the AGMs by flow cytometry. Although the percentage of non-HECs (cKit^−^CD45^−^CD31^+^Runx1-GFP^−^) was similar between the groups (Figure S2A), the percentage of Runx1-GFP^+^ HEC/EHT cells (cKit^−^CD45^−^CD31^+^Runx1-GFP^+^) was significantly lower in the KO AGM (Figures S2B and S2C), suggesting a role for PDGFRβ in HEC specification. Interestingly, the percentage of CD31^+^ckit^+^ IAHC cells in the KO was unchanged (Figures S2D and S2E). However, when we compared IAHC size, we found that the average IAHC area in the KO DAs was 1.7-fold higher than the WT (from 65.4 ± 11.7μm^2^ to 110.1 ± 2.3 μm^2^) (Figure 2C, left). In addition, a subset of KO IAHCs shows much larger areas (>200 μm^2^) than that the WT (Figure 2C, right). In contrast, the total number of IAHCs was on average 2-fold lower in the KO DAs (66 in the WT versus 33 in the KO). These data suggest that PDGFRβ-KO DAs produce fewer but larger clusters, leading to a normal number of CD31^+^ckit^+^ cells per DA. We next explored whether the effect of PDGFRβ deletion in HSPCs could be due to changes in the HSPC niche and/or to a developmental contribution of PDGFRβ^+^ cells to the HSPC pool.

### The genetic program of the PDGFRβ-KO AGM niche is altered

We examined the impact of PDGFRβ deletion on the hematopoietic niche by scRNA-seq of PDGFRβ-KO E11 AGMs (Figure S3A). Like in the WT AGM, we first defined the different populations using known genes. It was not possible to identify DP (PDGFRβ^+^NG2^+^) and PDGFRβ-S (PDGFRβ^+^NG2^−^) cells in the KO datasets due to *Pdgfrb* deletion (Figure S3A). Other cell types identified in the WT AGM were present in the AGM KO datasets, but we found significant changes in the proportions of MP, Ery/EryP, EC, SNS, clusters 7–10 altogether, and SkMPs (Figure S3B). We also observed a 1.5-fold decrease in the percentage of HEC/EHT (cluster 5; Figure S3B), supporting our flow cytometry findings (Figure S2C).

We next explored transcriptomic changes in the perivascular cells of WT and KO AGMs. Since DP and PDGFRβ-S perivascular cells were identified based on PDGFRβ expression, the absence of these cells in the KO AGM could be due to an impaired development of these subsets, or just reflect a loss of PDGFRβ expression. To test this, we stained and imaged whole-mount DAs and cryosections. While NG2 expression around the notochord appears similar, NG2 expression in perivascular cells is lower compared to the WT (Figures S3C–S3E). The expression of CD31 in ECs also appeared downregulated in the KO DA (Figures S3C and S3D). Interestingly, α-smooth muscle actin-positive (αSMA^+^) cells can be observed in both WT and KO AGM (Figure S3E). These data suggest that at least some PCs/VSMCs remain in place in the absence of PDGFRβ, although changes in their phenotype, gene profile, and function may exist.

To locate the equivalent of WT DP cells in our KO datasets, we attempted to identify a new gene that could replace *Pdgfrb* as a marker of DP cells in the WT population. Based on our imaging data, at least some WT DP cells become NG2-S in the KO, and thus, we searched for a gene expressed in WT DP cells but absent in WT NG2-S. Within the DEGs, *Rgs5* was the best candidate (Figure 1D), being expressed in 52% of DP cells and 13% of WT NG2-S cells (Table S4). Because we were unable to identify a completely overlapping replacement for *Pdgfrb* to analyze distinct KO AGM perivascular cell subsets, we defined the cluster containing all PDGFRβ^+^ cells in the WT as the *Rβ-niche*, and compared it against the corresponding cluster in the KO (Figure 2D, circle).

Most genes previously associated with hematopoietic support in AGM stromal cell lines (Charbord et al., 2014) were significantly downregulated in the *Rβ-niche* KO cluster (Figure S4A; Table S4). In the WT, we found that most genes are highly and mainly expressed by PCs/VSMCs (DP) and PDGFRβ-S cells (Figure S4A), suggesting that PDGFRβ^+^ cells play a role in the support of AGM HSPCs. To further understand the role of PDGFRβ in hematopoietic development, we performed an overrepresentation analysis on genes significantly downregulated in the *Rβ-niche* KO AGM cluster (Figure 2E). Several Gene Ontology (GO) biological processes were significantly overrepresented in these genes and downregulated in the KO perivascular niche, including regulation of stem cell differentiation, osteoblast differentiation, transforming growth factor β (TGF-β) receptor signaling, and mesenchymal cell differentiation. We found that *Bmp4*, a known regulator of hematopoiesis in the AGM (Bhatia et al., 1999; Chadwick et al., 2003; Crisan et al., 2015, 2016; Durand et al., 2007; McReynolds et al., 2007; Wilkinson et al., 2009) and a downstream target gene of PDGFRβ signaling (Nagel et al., 2019), was the top significantly downregulated gene encoding a secreted molecule associated with the mesenchymal cell differentiation GO term (Figure 2F; Table S4). Other genes involved in the bone morphogenetic protein (BMP) and TGF-β signaling, including ligands, receptors, and intracellular transducers but not BMP inhibitors, were significantly downregulated in the KO *Rβ-niche* (Figure S4B; Table S4). Genes associated with *Wnt* and *Notch* signaling pathways, which are essential for definitive hematopoiesis in the AGM (Bertrand et al., 2010; Bigas and Espinosa, 2012; Burns et al., 2005; Clements et al., 2011; Gering and Patient, 2010; Goessling et al., 2009; Kumano et al., 2003; Ruiz-Herguido et al., 2012), were also significantly downregulated in the KO *Rβ-niche* (Figures 2F, S4C, and S4D; Table S4).

Other genes implicated in mesenchymal cell differentiation GO term and reported to regulate embryonic hematopoiesis included *Hif1a*, *Ednra*, *Aldh1a2*, *and Kitl* (Azzoni et al., 2018; Chanda et al., 2013; Crosse et al., 2020; Imanirad et al., 2012; Lim et al., 2017). Interestingly, genes associated with adult hematopoiesis such as *Col1a2*, *Cited-2*, *Cxcl12*, *Nes*, and *Mcam* (Figures 2F and S4A), as well as *Kitl* and *Jag1* (Corselli et al., 2013; Ding et al., 2012; Greenbaum et al., 2013; Isern et al., 2013; Kranc et al., 2009; Kunisaki et al., 2013; Mendez-Ferrer et al., 2010; Omatsu et al., 2010; Sacchetti et al., 2007), were also significantly downregulated in the KO *Rβ-niche*, suggesting that PDGFRβ^+^ cells potentially support adult-type HSPCs.

To predict ligand-receptor interactions between ECs or IAHCs and niche cells, we applied NicheNet to our WT scRNA-seq data, focusing on mesenchymal differentiation GO term genesets in PCs/VSMC (DP) cells. NicheNet analysis first provided us with a list of ligands expressed in DP cells ranked by predicted ligand activity, where *Bmp4* scored the highest (Table S4). Moreover, the highest scoring predicted interaction we found was between *Bmp4*, expressed by DP cells, and *Bmpr2* found in both EC (Figure 2G) and IAHC (Figure 2H) clusters, followed by *Tgfb1*:*Tgfbr1/2* (Figures 2G and 2H). *Lrp6* in EC or HSPC also has a relatively strong predicted interaction potential with *Dkk2* and/or *Wnt5a* in DP cells. These data suggest that Bmp4 is one of the main secreted molecules mediating interactions between vascular cells and the niche in the AGM.

### PDGFRβ^+^ cells are required in the AGM niche to sustain hematopoiesis

**Figure 3:**
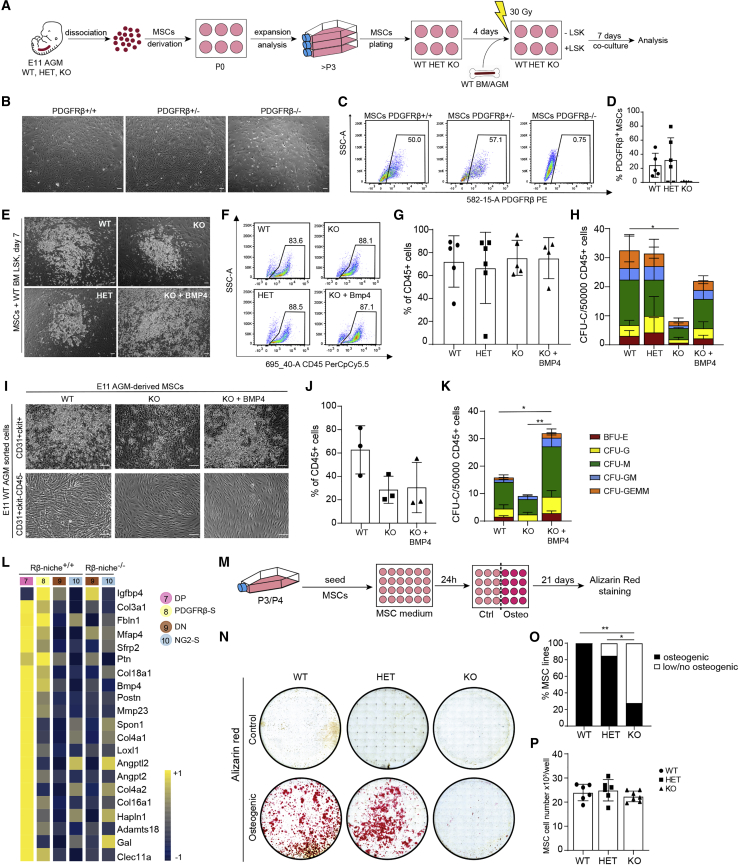
PDGFRβ^+^ MSCs are essential for hematopoiesis (A) Schema of co-cultures AGM MSCs and BM LSKs or AGM HSPC/ECs. (B–D) Images of P3 MSCs; 100μm. Example of flow cytometry plots (C) and histograms (D) showing PDGFRβ expression in MSCs; error bars: SD. (E) Images of PDGFRβ WT and mutant E11 AGM MSCs co-cultured with BM LSKs; 100μm. (F) Gating showing the percentage of CD45^+^ cells derived from MSC-LSK co-cultures. (G and H) Percentage of CD45^+^ cells 7 days post-co-culture (G) and CFU-C numbers obtained from co-cultures (H) (n = 5/6/5/4 MSC lines) (see Table S5); error bars: SD; ∗p < 0.05. (I) Images of MSCs co-cultured with WT E11 AGM-derived HPSCs and ECs; 100μm. (J) Percentage of CD45^+^ cells 7 days post-co-culture; error bars: SD. (K) CFU-Cs obtained from co-cultures with E11 AGM MSCs; error bars: SD; ∗p < 0.05, ∗∗p < 0.01. (I–K) n = 3/3/3 MSC lines (see Table S5). (L) Expression of osteogenic genes in WT/KO *Rβ-niche* (see Table S4). (M) Osteogenic assay. (N) Example of alizarin red staining of PDGFRβ WT, HET, and KO MSCs. (O) Percentage of osteogenic MSC lines. WT: 6/6; HET: 6/7; and KO: 2/7. ^∗^p = 0.015, ^∗∗^p = 0.006, z test for proportions. (P) Numbers of MSCs at day 0 of osteogenic assay (WT/HET/KO = 6/6/7); error bars: SD.

Since PDGFRβ deletion seems to affect mesenchymal cell differentiation (Figure 2E), and AGM mesenchymal stem/stromal cells (MSCs) are known to support hematopoiesis (Oostendorp et al., 2002a), we derived MSCs from E11 PDGFRβ WT and KO AGMs (Figure 3A). These MSC cultures did not contain HSPCs or ECs as they did not express CD31 nor CD45 and did not form CFU-Cs (data not shown). They also exhibited the expected MSC morphology, although some differences were observed in the KO (Figures 3B–3D).

To assess their hematopoietic support, we cultured MSCs from each genotype as monolayers, irradiated and co-cultured them with WT BM Lin^−^Sca1^+^Kit^+^ (LSKs) (Figure 3A). Large hematopoietic colonies were found after 7 days in co-cultures with all three MSC genotypes (Figure 3E). Although we found a similar percentage of CD45^+^ cells (Figures 3F and 3G), PDGFRβ-KO MSC co-cultures yielded a significantly lower total number of CFU-Cs (Figure 3H; Table S5). Since we have previously shown that BMP4 controls at least a subset of HSPCs in the E11 AGM (Crisan et al., 2015, 2016) and *Bmp4* is significantly downregulated gene in the KO *Rβ-niche* cluster, we performed co-culture experiments in the presence of BMP4. The addition of BMP4 to KO co-cultures increased the numbers of CFU-Cs (Figure 3H; Table S5). To test whether AGM-derived HSPCs were also affected in co-cultures with AGM KO MSCs, we sorted CD31^+^ckit^+^ (IAHC containing HSPCs) and CD31^+^ckit^−^CD45^−^ (ECs) from E11 WT AGMs and co-cultured them with irradiated WT and KO AGM-derived MSCs. Hematopoietic cells were detected only in co-cultures containing CD31^+^ckit^+^ cells, and their numbers were lower in co-cultures with KO MSCs (Figure 3I). This was confirmed by flow cytometry, although the difference was not significant (Figure 3J). The total number of CFU-Cs derived from KO MSC co-cultures was also lower (Figure 3K), with a significant decrease in immature CFU- granulocyte/erythrocyte/macrophage/megakaryocyte (GEMM) and complete absence of BFU-E colonies (Table S5). Interestingly, exogenous BMP4 was able to not only rescue HPSCs, but also expanded them (Figure 3K; Table S5). Altogether, these data demonstrate that PDGFRβ^+^ cells play a role in the AGM niche to sustain hematopoiesis, likely through the involvement of BMP4.

### PDGFRβ-KO AGMs have an altered osteogenic transcriptome profile and differentiation potential

The regulation of osteoblast and MSC differentiation biological processes were among the GO terms significantly overrepresented among genes significantly downregulated in the *Rβ-niche* KO AGM cluster (Figure 2E). Since AGM MSCs are known to be osteogenic (Durand et al., 2006; Mendes et al., 2005), we examined the osteogenic potential of AGM-derived PDGFRβ KO MSCs. Genes characteristic of both osteoblasts and osteocytes in mice (Paic et al., 2009) were significantly downregulated in the KO *Rβ-niche* (Figure 3L; Table S4) and were mainly expressed by the WT *Pdgfrb*-expressing clusters 7 and 8. To test whether their osteogenic potential was affected, multiple MSC lines were cultured in osteogenic or control media and stained with alizarin red (Figure 3M). While all of the WT MSCs and the majority of HET MSC lines tested were osteogenic (6/6 and 6/7, respectively), most KO MSC lines showed low/no alizarin red staining (5/7) (Figures 3N and 3O). MSC numbers on day 0 were similar between groups (Figure 3P).

We next questioned whether the defect in HSC support in culture with KO MSCs was due to the absence of the supportive perivascular cells normally found in the AGM or to the absence of PDGFRβ on their surface. Since these cells remain α-SMA^+^ in the KO AGM *in vivo*, we investigated whether α-SMA expression persists in KO-derived MSCs in culture. We confirmed that both WT and KO AGM MSCs express α-SMA (Figure S3F). We have previously shown that PCs/VSMCs are MSC precursors and that both pericytes and MSCs express CD146 (Crisan et al., 2008). In addition, a recent study showed that the layer of cells adjacent to midgestation DA in the mouse is CD146^+^NG2^+^ and marks VSMC precursors (Roostalu et al., 2018). We confirmed that CD146 is expressed in WT (CD31^−^) AGM perivascular cells and found that its expression persists in the KO AGM (Figure S3G) and on cultured MSCs (Figure S3H), suggesting that some perivascular cells that normally express PDGFRβ in the WT DA are still present in KO embryos, while missing the PDGFβ receptor. These data demonstrate that the loss of PDGFRβ alters the osteogenic potential of the AGM, and that PDGFRβ signaling plays a role in the AGM hematopoietic niche.

### Vascular and hematopoietic gene profile changes in the PDGFRβ-KO AGM

**Figure 4:**
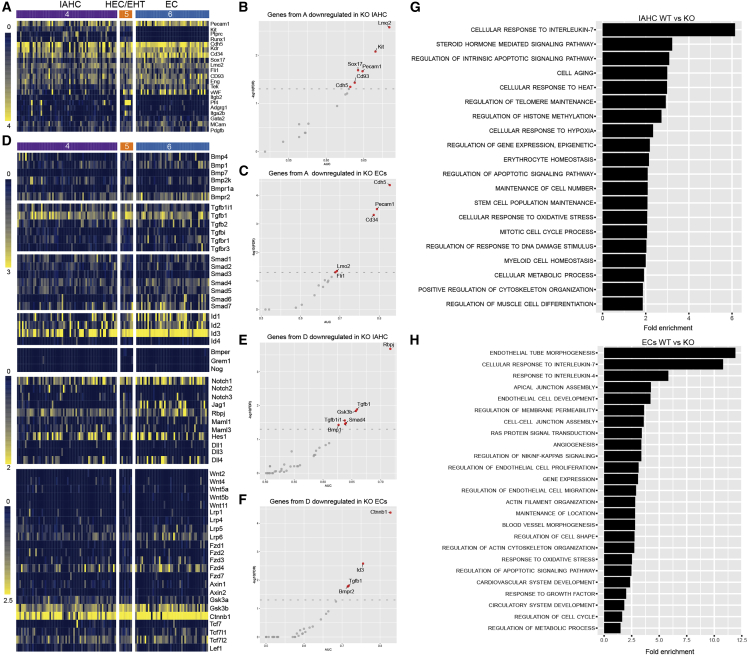
Transcriptomic differences by scRNA-seq between WT and KO EC, HEC/EHT, and IAHCs (A) Heatmap showing the expression of *Pecam1*, *Kit*, *Ptprc*, and *Runx1* identifying these clusters. (B and C) Scatterplots with genes from (A) that are downregulated in IAHC and ECs. (D) Genes associated with BMP/TGFβ, NOTCH, and WNT pathways in EC, HEC/EHT, and IAHCs. (E and F) Genes from (D) that are downregulated in IAHC and ECs. Red dots: significantly downregulated genes. (G and H) Selected biological processes significantly overrepresented in genes significantly downregulated in the KO IAHC (G) and in the KO ECs (H).

**Figure 5:**
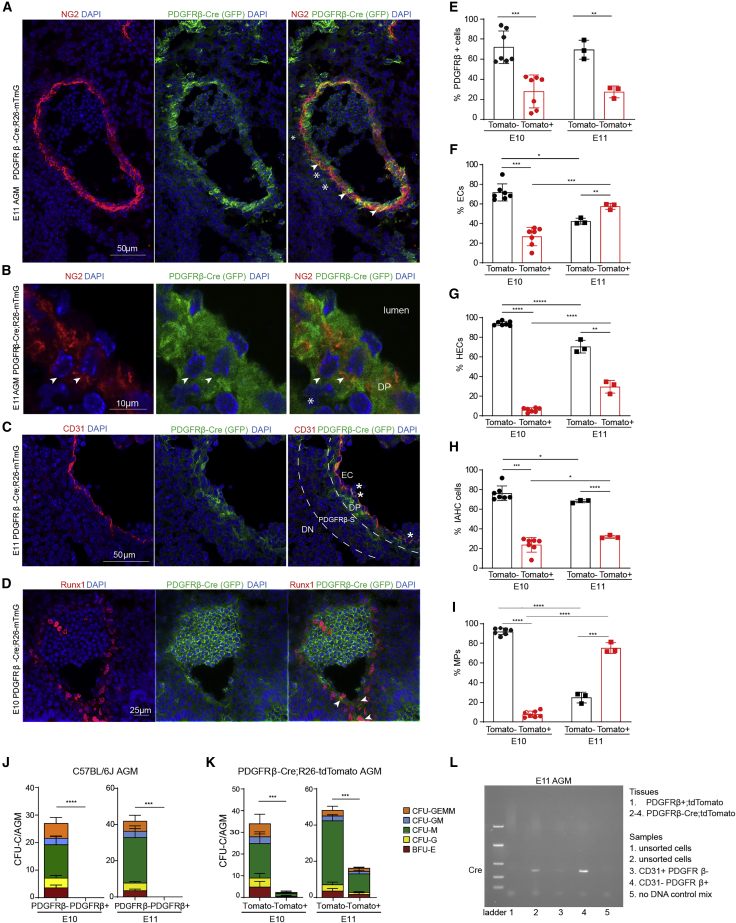
A subset of ECs, HEC/EHT, and IAHCs derived from PDGFRβ^+^ precursors (A–D) Immunohistochemistry of E11 PDGFRβ-Cre:mTmG AGM stained with NG2 (A and B) and CD31 (C) and at E10 with Runx1 (D). (A and B) Arrowheads: DP cells; stars: GFP^+^ mesenchymal cells. (C) Dashed lines: presumptive separation between DP, PDGFRβ-S, and DN layers; stars: hematopoietic CD31^+^GFP^+^ cells. (E–I) Flow cytometry analyses of E10 (n = 7) and E11 (n = 3) PDGFRβ-Cre; tdTomato AGMs, percentage of Tomato^+/−^ cells within (E) PDGFRβ^+^ cells, (F) EC (CD45^−^cKit^−^CD31^+^CD41^−^), (G) HEC/EHT-enriched population (CD45^−^cKit^−^CD31^+^CD41^+^), (H) IAHC/HSPC (CD31^+^cKit^+^), and (I) MP-enriched population (CD45^+^CD31^−^) live cells; error bars: SD; ∗p < 0.05, ∗∗p < 0.005, ∗∗∗p < 0.005, ∗∗∗∗p < 0.0005. (J) CFU-Cs on PDGFRβ^+/−^ cells sorted from E10 (n = 8) and E11 (n = 6) AGMs (see Table S6). E11 AGM were ckit^+^; error bars: SD; ∗∗∗p < 0.005, ∗∗∗∗p < 0.0005. (K) CFU-Cs on Tomato^+/−^ cells sorted from PDGFRβ-Cre; tdTomato E10 (n = 6) and E11 (n = 4) AGMs (see Table S7); error bars: SD; ∗∗∗p < 0.005. (L) PCR on E11 unsorted cells from PDGFRβ-Cre; tdTomato and +; tdTomato WT littermates heads and from sorted AGM cells to detect Cre recombinase expression (324 bp). Unpaired *t* tests or Mann-Whitney tests were used for the statistics.

We next investigated the effect of PDGFRβ loss on endothelial and hematopoietic cells. We confirmed the identity of ECs (*Pecam*^*+*^*Kit*^−^*Ptprc*^−^*Runx1*^−^), HEC/EHT (*Pecam1*^*+*^*Kit*^−^*Ptprc*^−^*Runx1*^*+*^), and IAHC (*Pecam1*^*+*^*Kit*^*+*^) cells in the WT AGM clusters (Figure 4A). The presence of *Cdh5*, *Kdr*, *CD34*, *Sox17*, and other genes further validate their cell identity (Figure 5A). Our datasets also confirmed that IAHC cells are heterogeneous, with a subset expressing *Ptprc* and/or *Runx1*, but also *Adgrg1* and *Gata2*. *Itga2b* (CD41), a gene upregulated in HEC/EHT and nascent HSCs (Boisset et al., 2010; Eilken et al., 2009; Fadlullah et al., 2022; Kissa and Herbomel, 2010; Mikkola et al., 2003; Zhu et al., 2020), was detected in a subset of cells within the IAHC population, as expected (Figure 4A). While *Itga2b* was absent in ECs, the HEC/EHT cell cluster was composed of both *Itga2b*^*+*^ and *Itga2b*^*low/*−^ cells (Figure 4A), indicating that some of these cells have started transitioning toward the hematopoietic program. Interestingly, *Pecam1* was significantly downregulated in both KO IAHCs and ECs (Figures 4B and 4C), in line with our imaging observations (Figures S3C and S3D). Other significantly downregulated genes in IAHCs were *Kit*, *Sox17*, *CD93*, and *Cdh5* (Figure 4B). Moreover, *Lmo2*, a transcription factor critical for normal hematopoietic and endothelial development (Landry et al., 2005), was significantly downregulated in both IAHCs and ECs (Figures 4B and 4C). Since genes involved in BMP, Wnt, and Notch signaling were downregulated in the KO *Rβ-niche* (Figures S4B–S4D), we examined related signals and receptors in EC, HEC/EHT, and IAHC clusters. *Bmpr2* was present in IAHC, EC, and HEC/EHT clusters (Figure 4D), but significantly downregulated in ECs (Figure 4F), suggesting that BMP signaling is altered. Other signaling molecules such as *Rbpj*, *Tgfb1*, *Smad4*, *Gsk3b*, and *Ctnnb1* were significantly downregulated in IAHCs and *Id3* and *Tgfb1* in ECs (Figures 4D–4F). No transcriptional changes were found in the KO HEC/EHT, although this finding is inconclusive due to the low number of cells captured.

To further understand the role of PDGFRβ in hematopoiesis, we performed an overrepresentation analysis on genes significantly downregulated in IAHC and EC KO AGM populations (Figures 4G and 4H). Several biological processes were significantly overrepresented in these genes and downregulated in KO IAHCs, including cellular response to interleukin-7 (IL-7) and to hypoxia (Figure 4G). Genes associated with biological processes including EC development, angiogenesis, cell migration, and response to growth factors were significantly altered in the EC cluster (Figure 4H); some processes were common to both clusters, including regulation of apoptosis, metabolic process and response to oxidative stress, cell cycle, and actin cytoskeleton organization (Figures 4G and 4H). Altogether, these data show that both hematopoietic and vascular cell compartments are transcriptomically affected in the AGM in the absence of PDGFRβ.

### PDGFRβ cells labeled in the early embryo contribute to the AGM EC pool

**Figure 6:**
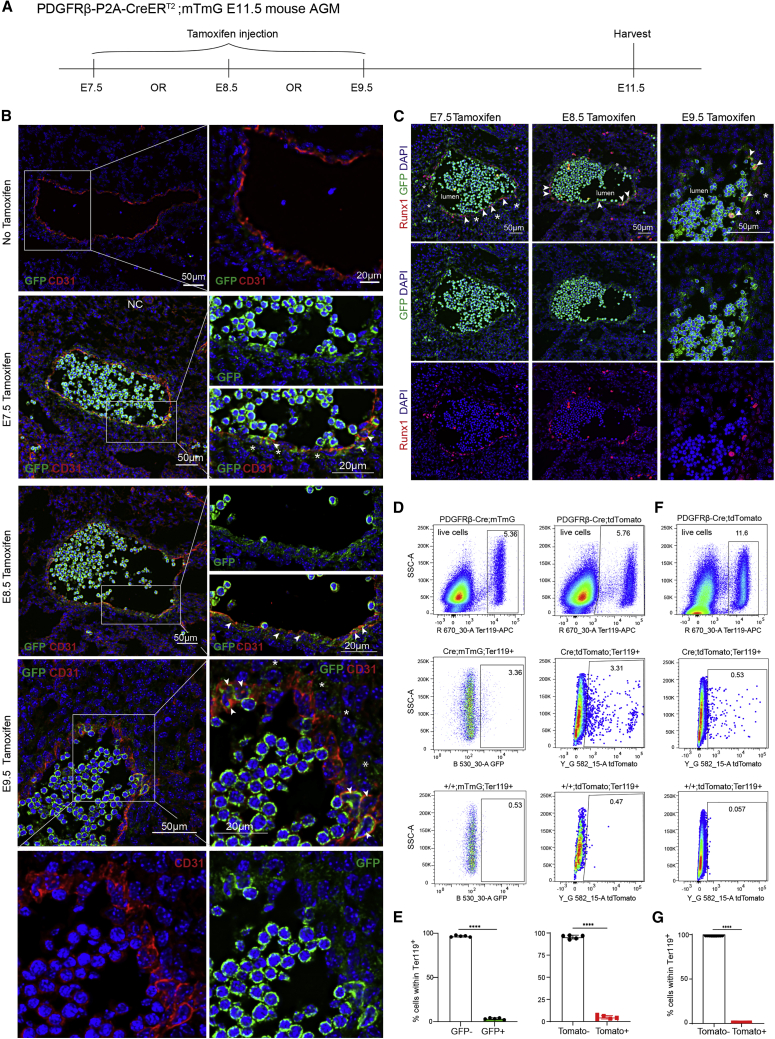
PDGFRβ^+^ cells from E7.5–E9.5 mouse embryo contribute to peri/vascular/hematopoietic lineages present in the E11.5 AGM (A) PDGFRβ-Cre-derived cell tracing. (B and C) Immunohistochemistry on PDGFRβ-P2A-CreERT2; mTmG E11 AGMs showing CD31 and GFP (B) and Runx1 and GFP (C). (D–G) Flow cytometry plots (D and F) and quantification (E and G) of GFP or Tomato expression within Ter119^+^ cells from E11 AGM (D and E) (n = 5) and YS (n = 9) (F and G); error bars: SD; ^∗∗∗∗^p < 0.0001, unpaired *t* test.

Although we found a direct involvement of PDGFRβ^+^ MSCs in the HSPC niche, the decrease in HSPCs could also be due to the loss of PDGFRβ expression in HSPCs or in their precursors. To investigate this, we lineage traced PDGFRβ^+^ cells and their progeny using *PDGFRβ-Cre* and *R26-mTmG* mice. We confirmed the presence of GFP in perivascular cells in E10 and E11 AGMs (Figures 5A–5D). GFP is expressed by PCs/VSMCs (NG2^+^) that are in close contact with ECs (NG2^−^) (Figures 5A and 5B, arrowheads) as well as in PDGFRβ-S (NG2^−^) cells (Figures 5A and 5B, stars). Importantly, we found some CD31^+^GFP^+^ cells (Figure 5C) and Runx1^+^GFP^+^ cells (Figure 6D, arrowheads) suggesting that some ECs and some HSPCs derive from PDGFRβ^+^ precursors. Flow cytometry analyses of PDGFRβ-Cre; tdTomato AGMs confirmed these observations (Figures 5E–5H and S5A–S5E). We used CD41 to distinguish between EC and HEC/EHT cells, and showed that on average, 26.80% and 57.60% of ECs (CD41^−^) express tdTomato at E10 and at E11, respectively (Figures 5F and S5C), whereas 5.93% and 29.57% of HEC/EHT cells express tdTomato at E10 and E11 (Figures 5G and S5C). We also found that 23.76% and 31.87% of CD31^+^ckit^+^ IAHC cells were Tomato^+^ at E10 and E11, respectively (Figures 5H and S5D). Moreover, a subset of IAHC cells expressed CD41 and both CD41^+^ and CD41^−^ IAHCs express tdTomato (Figure S5D). We next examined the expression of tdTomato in MPs (CD45^+^CD31^−^) and found that a majority express tdTomato in E11 AGM (Figure 5I), while only a small fraction expresses it at E10 (Figures 5I and S5E). These data suggest that a subset of ECs, HEC/EHT cells, IAHC cells, and MPs either express PDGFRβ or derive from PDGFRβ^+^ precursors.

Although we found no overlap between CD31 and PDGFRβ by immunohistochemistry (Figure 1A), we examined whether there was an overlap between *Pdgfrb*, *Pecam1*, and *Ptprc* gene expression in our WT scRNA-seq datasets. *Pdgfrb* was absent in the EC cluster (Figure S5F) and rare cells were positive for *Pdgfrb* in clusters 4 and 5. However, the expression of *Pdgfrb* was low and the doublet score indicates that they are likely doublets (Figure S5F). A few DP and PDGFRβ-S cells found to co-express *Pecam1* and/or *Ptprc* at a low level also have a high doublet score (Figure S5F). These data suggest that *Pecam1* and *Ptprc* are not expressed in DP and PDGFRβ-S populations and *Pdgfrb* is not expressed in HEC/EHT and IAHC cells.

To test whether PDGFRβ is expressed in HSPCs, we performed CFU-C assays on E10 and E11 AGM PDGFRβ^+^ (and PDGFRβ^−^ cells) sorted cells (Figures S5G and S5H). Only the PDGFRβ^−^ cell fraction formed colonies (Figure 5J; Table S6), suggesting that AGM HSPCs do not express PDGFRβ. To test whether PDGFRβ cells are precursors of HSPCs, we sorted tdTomato^+^ and tdTomato^−^ cells from PDGFRβ-Cre; tdTomato E10 and E11 AGMs (Figure S5I) and seeded in methylcellulose. At E10, most colonies derived from tdTomato^−^ cells (Figure 5K; Table S6), whereas at E11, one-third of the colonies were tdTomato^+^, demonstrating that a subset of HSPCs derive from PDGFRβ-Cre precursors. We confirmed that Cre recombinase was expressed in the PDGFRβ^+^CD31^−^ sorted cells and was low/negative in the CD31^+^PDGFRβ^−^ sorted cells (Figure 5L). Together, these data show that PDGFRβ marks a subset of HSPC precursors, but not functional HSPCs, suggesting that there are at least two different origins to AGM-derived HSPCs.

To understand the difference between Tomato^+^ (T+) and Tomato^−^ (T-) IAHC/HSPCs, we compared them by flow cytometry. A recent study found that IAHCs contain 2 populations of CD31^+^ cells (CD31^hi^ and CD31^lo^) that are transcriptomically and functionally distinct, with CD31^hi^ being enriched in transplantable HSCs (Vink et al., 2020). Here, we found that T- IAHCs are mainly CD31^lo^ and contain significantly more CD45^+^ cells than the CD31^hi^ cell population, suggesting a more mature phenotype (Figures S6A–S6E). The T+ IAHCs have similar frequencies of CD31^hi^ and CD31^lo^ cells, and more CD31^lo^ also express CD45 (Figures S6A–S6E). Interestingly, T+ IAHC cells are significantly enriched in CD31^hi^ cells when compared to T- IAHC cells (Figure S6D), whereas T- IAHC cells are significantly enriched in CD31^lo^ cells when compared to T+ IAHC cells (Figure S6E).

Further analysis of our scRNA-seq dataset similarly suggests that IAHC/HSPCs are divided in 2 subclusters: *Pecam1* high (*CD31*^*hi*^) and *Pecam1* low (*CD31*^*lo*^) (Figure S6F). *CD31*^*hi*^ cells are enriched in genes including *Kdr*, *Cdh5*, *Sox7*, *Sox17*, and *Sox18*; interestingly, they are also enriched in *Bmpr2* (Figures S6F and S6G; Table S4). In contrast, *CD31*^*lo*^ are enriched in genes, including *Il7r*, while genes such as *Kit* and *Hif1α* show similar expression levels in both *CD31*^*hi*^ and *CD31*^*lo*^ subclusters. We next performed differential expression and subsequent enrichment analysis to compare *CD31*^*hi*^ and *CD31*^*lo*^ subclusters, and found that *CD31*^*hi*^ is significantly enriched in genes involved in GO biological processes that include the regulation of the cell cycle, BMP/TGF-β pathways, NOTCH and WNT signaling, and response to hypoxia (Figure S6H). In contrast, the *CD31*^*lo*^ subcluster is enriched in genes involved in the regulation of ILs such as IL-1, -6, -7, -10, and -12, immune response, glyceraldehyde-3-phosphate metabolic process, and others (Figure S6I). Importantly, the frequency of *CD31*^*hi*^ IAHC decreases in the KO AGM, from 0.41% to 0.32%, whereas the *CD31*^*lo*^ IAHC proportion remains unchanged, suggesting that at least some T+ IAHC/HSPCs are found within the *CD31*^*hi*^ subcluster. In addition, there are 2,179 genes that are downregulated in the *CD31*^*hi*^ subcluster between WT and KO and these include *Pecam1*, *Cdh5*, *Kit*, *Sox7*, *Sox17*, *Sox18*, and *Hif1α* (Table S4). Interestingly, *Bmpr2* was enriched in the *CD31*^*hi*^ subcluster (Table S4), but not significantly downregulated in the KO (Table S4), suggesting that the impaired HSPC defect we found in the KO AGM is due at least in part to low BMP4 released by the niche cells, in line with our scRNA-seq datasets (Figure 2) and functional assays (Figure 3). Together, these data suggest that the two phenotypically distinct populations of IAHC/HSPCs may also differ transcriptomically, although it remains unclear whether they fully overlap at the protein and transcript levels.

To determine the onset of PDGFRβ-Cre expression in the AGM, we used *PDGFRβ-P2A-CreER*^*T2*^ *and R26-mTmG* mice. Pregnant dams were injected with a single dose of tamoxifen at either E7.5, E8.5, or E9.5 and embryos were harvested at E11.5 (Figure 6A). Numerous CD31^+^ cells lining the DA expressed GFP after E7.5 and E8.5 injections (Figure 6B, arrowheads), and fewer expressed GFP after E9.5 injections. In addition, some GFP^+^ perivascular mesenchymal cells were observed (Figures 6B and 6C, stars). We also observed Runx1^+^GFP^+^ cells in some vascular, perivascular, and hematopoietic cells (Figure 6C, arrowheads), suggesting that IAHCs are heterogeneous.

To investigate whether erythroid cells in the lumen are GFP^+^ or simply autofluorescent, we performed flow cytometric analyses of E11 AGMs harvested from both PDGFRβ-Cre; mTmG and PDGFRβ-Cre; tdTomato embryos using Ter119, an erythrocyte differentiation marker. We found that on average, 3.4% and 4.7% of Ter119^+^ cells are GFP^+^ and tdTomato^+^, respectively (Figures 6D and 6E), and most cells in sections also fluoresce in the CD31 channel (Figure 6B), suggesting that the majority of the cells in the lumen on sections are autofluorescent, as has been found in another study (Yzaguirre et al., 2015). We next looked at the PDGFRβ-Cre contribution to YS erythroid cells and found only 0.16% Ter119^+^Tomato^+^ cells in the E11 YS (Figures 6F and 6G), suggesting either a limited contribution of PDGFRβ-derived erythroid cells from the YS or circulating AGM-derived cells. These data are in line with a study showing that PDGFRβ-Cre does not label hematopoietic cells nor HECs in the E9 and E10 YS (Ulvmar et al., 2016). Altogether, these findings show that, in addition to relevant perivascular cells in the AGM hematopoietic niche, PDGFRβ-Cre labeling also identifies ECs and possibly HECs in the AGM.

### PDGFRβ-derived HSPCs persist during hematopoietic ontogeny

To test whether PDGFRβ-derived HSPCs persist at later stages of development and adulthood, we examined E14 FL and adult BM for PDGFRβ expression. Very rare PDGFRβ^+^ cells were found at E14, confirming a previous report (Kaminski et al., 2001). Sorted PDGFRβ^+^ cells from E14 FL and adult BM failed to form CFU-Cs (Table S7), suggesting that HSPCs in those organs do not express PDGFRβ.

**Figure 7:**
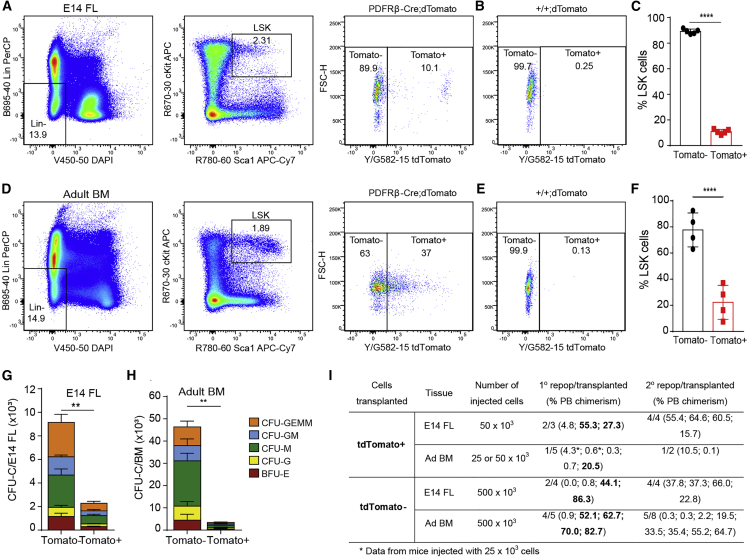
A subset of E14 FL and adult BM LSKs derived from PDGFRβ^+^ precursors (A–H) Analysis of Tomato^+/−^ LSKs from PDGFRβ-Cre; tdTomato and PDGFRβ^+/+^; tdTomato E14 FL littermates (A and B) and adult BM (D and E). Percentage of Tomato^+/−^ within LSKs per E14 FL (n = 5) (C) and adult BM (n = 4) (F). CFU-Cs from sorted Tomato^+/−^ cells E14 FL (n = 5) (G) and adult BM (n = 4) (H); error bars: SD; ^∗∗^p < 0.01, ^∗∗∗∗^p < 0.0001 (see Table S7). (I) Transplantation of Tomato^+/−^ cells sorted from E14FL and adult BM into primary (1°) and secondary (2°) recipients.

Interestingly, we found that a fraction of LSKs from PDGFRβ-Cre; tdTomato E14 FL and adult BM express tdTomato (Figures 7A–7F). We next performed CFU assays and transplantations on sorted tdTomato^+^ and tdTomato^−^ cells from both organs. CFU-Cs were detected in both fractions of the FL and BM (Figures 7G, 7H, and S7A–S7D; Table S7). Primary and secondary transplantations confirmed the presence of HSCs in both fractions (Figures 7I, S7E, and S7F). These data demonstrate that both developing and adult hematopoietic cells derive in part from PDGFRβ^+^ embryonic precursors.

## Discussion

The primary objective of this study was to identify and characterize the perivascular cells that constitute the HSPC microenvironment in the mouse AGM and to reveal the specific cells that play a supportive role during HSPC generation. Our results revealed two populations of cells surrounding the DA that express PDGFRβ, are enriched in HSC-supportive genes, and have osteogenic potential. This led us to hypothesize that PDGFRβ^+^ perivascular cells surrounding the DA play a role in HSPC development in the AGM. Indeed, in the absence of PDGFRβ, AGM HSPC activity was significantly reduced, suggesting a key role for PDGFRβ^+^ cells in the regulation of HSPC specification. We discovered that PDGFRβ has a dual role. First, PDGFRβ^+^ AGM niche cells support definitive HSPCs, and the loss of PDGFRβ greatly reduces HSPC activity. Second, PDGFRβ^+^ precursor cells (transiently expressing PDGFRβ) in the early embryo give rise to a subset of ECs and HSPCs in the AGM that persist into adulthood.

### Developmental expression of *Pdgfrb* and lineage tracing of HSPCs

Using lineage tracing, we found that that a subset of AGM HSPCs and their endothelial precursors derive from a population of PDGFRβ^+^ cells in early development. A similar result was published (Zovein et al., 2008), where it was found that the constitutive *SM22-Cre* marked ECs and hematopoietic cells, but the tamoxifen-inducible *iSM22-Cre* (injected at E9.5) excluded them. These data suggest that a population of early mesoderm precursor cells can later develop into endothelial, hemogenic endothelial, and hematopoietic cells. In addition, a study in the chick embryo showed that both aortic ECs and mesenchyme have a splanchnopleuric origin (Richard et al., 2013). In a recently published single-cell atlas of mouse embryonic development, we explored the expression of *Pdgfrb* in the mouse embryo between E6.5 and E8.5 (Pijuan-Sala et al., 2019) and found that, in addition to the mesoderm and mesenchymal cells, *Pdgfrb* is also expressed in a subset of hemato-ECs. PDGFRβ was previously shown to promote early EC differentiation in mice (Rolny et al., 2006). This group described a population of hemangio-precursors that co-expressed PDGFRβ, vascular endothelial growth factor receptor 2 (VEGFR2), and CD41 in differentiating embryonic bodies that give rise to both ECs and hematopoietic cells in culture (Rolny et al., 2006). In line with these studies, besides labeling the subaortic mesenchyme in the AGM, we found that PDGFRβ^+^ cells from E7.5 to E9.5 of development are also able to give rise to hemogenic cells/ECs. However, the AGM subaortic mesenchyme, expressing PDGFRβ, does not form hematopoietic colonies *in vitro*, further supporting the study by Zovein et al. (2008).

Interestingly, one study showed that HSPC activity in the PDGFRβ-KO E14 FL is similar to that in the WT, both *in vitro* and *in vivo* (Kaminski et al., 2001). Our study demonstrates that only a subset of E14 FL HSPCs derives from PDGFRβ^+^ cell precursors, suggesting that the remaining HSPCs are sufficient to compensate for the absence of PDGFRβ-derived HSPCs in the KO mice and support the finding that HSPCs are heterogeneous (Crisan and Dzierzak, 2016).

### Other niche molecules regulating hematopoietic development

We found that some developing/embryonic and adult HSPCs are controlled by BMP4 in co-culture experiments. We also confirmed that a subset of AGM EC and IAHC cells express BMP receptors, as previously shown (Crisan et al., 2015, 2016), and therefore can respond to BMP ligands. Interestingly, we found that *Bmp4* was downregulated in the KO *Rβ-niche*, and so was one of its receptors (*Bmpr2*) in the KO EC cluster. This impaired HSPC activity/number could be attributed, at least in part, to impaired HEC specification. We found fewer HEC/EHT cells, IAHCs, and CFU-Cs in the PDGFRβ-KO AGM which correlates with the absence of PDGFRβ-derived cell precursors. The remaining (non-PDGFRβ-derived) HEC/EHT cells were able to give rise to functional HSPCs and larger IAHCs. In line with our data, large IAHCs and fewer IAHCs and transplantable HSCs were also reported in *Svep1*^*−/−*^ E10.5 AGMs (Yvernogeau et al., 2020). We found no changes in *Svep1* in the *Rβ-niche* (not shown), likely due to the heterogeneity of the cells captured. Large IAHCs were also observed upon Dll4 blockage in the DA (Porcheri et al., 2020). While *Dll4* expression did not change in the PDGFRβ-KO scRNA-seq, we found a downregulation of Notch signaling in the *Rβ-niche*, suggesting that PDGFRβ^+^ cells in the AGM hematopoietic niche may control the IAHC size by regulating Notch signaling.

### Pericyte precursors and the osteogenic and hematopoietic niche

We have previously identified pericytes as precursors of functional MSCs. They are able to differentiate into bone (Crisan et al., 2008) and PDGF-B/PDGFRβ signaling in cooperation with BMPs and other known pro-osteogenic growth factors, control the pericyte-MSC-osteoblast differentiation axis (Caplan and Correa, 2011). Our findings showing that genes associated with MSC differentiation and osteogenic development were significantly downregulated in the KO *Rβ-niche*, together with a lack of calcium deposition *in vitro*, are strongly supportive. Our data also show that genes enriched in multipotent stromal cells (e.g., *Nrp1*, *Yap1*, *CD248*) compared to those less potent (Rosu-Myles et al., 2012), were significantly downregulated in the KO *Rβ-niche*. However, the perivascular-MSC-hematopoietic support axis *in vivo* in which PDGFRβ is a central network controller has not been described so far in the mouse. Our study shows that PDGFRβ^+^ cells represent the major source of BMP, NOTCH, WNT, and KIT ligands and other molecules previously identified as regulators of hematopoietic development in the AGM niche. While these molecules are mainly enriched in DP (PC/VSMC) cells, many are found in both DP and PDGFRβ-S in our scRNA-seq datasets, including *Bmp4*. As we were unable to culture DP cells, we are unable to conclude which population has a better capacity to support hematopoiesis. Moreover, the perivascular niche in the AGM is heterogeneous and complex, making it unlikely that the hematopoietic defects we found in the KO were exclusively due to the loss of MSC potential or BMP signals. Other populations had altered transcriptomes, including MP, Ery/EryP, SNS, and SkMP, some of which were previously implicated in the AGM niche (Fitch et al., 2012; Mariani et al., 2019). In zebrafish, 48% of *pdgfrb* morphants show a neural crest migration defect (Damm and Clements, 2017), but we found no differences in neural crest migration, as neural crest-derived TH^+^ cells were present in the KO DA (not shown).

In conclusion, the identification of a developmental link between PDGFRβ^+^ cells and embryonic and adult HSPCs provides a basis for HSPC heterogeneity. Furthermore, the requirement of PDGFRβ expression in AGM perivascular cells provides additional cues for the controlled production and maintenance of HSPCs *in vitro*. Future projects aim to dissect the cooperation between perivascular cells and other cells of the niche. Importantly, investigating the molecular and functional differences between PDGFRβ-derived and non-PDGFRβ-derived HSPCs, will add valuable insight to their roles in homeostasis, aging, and disease.

### Limitations of the study

Performing scRNA-seq of the full AGM allowed us to capture many populations of cells, but rare populations are poorly represented. We were also unable to find a gene fully overlapping PDGFRβ^+^ cells in the KO, and therefore we analyzed the *Rβ-niche*, which contains all of the relevant cells, but also some DN cells. Lastly, because the recombination efficiency is not 100%, some of the results may be underestimated.

## STAR★Methods

### Key resources table

**Table:** 

REAGENT or RESOURCE	SOURCE	IDENTIFIER
**Antibodies**		

Rat anti-mouse cKit	BD Bioscience	Cat# 553352; RRID:AB_394803
Rabbit polyclonal NG2	Millipore	Cat# ab5320; RRID:AB_91789
Rat anti-mouse biotinylated CD31	BD Pharmingen	Cat# 553371; RRID:AB_394817
streptavidin Cy3	Sigma	Cat# S6402; RRID:AB_2920525
Rat anti-mouse CD146-AF488	Biolegend	Cat# 134707; RRID:AB_10641136
Rat anti-mouse NG2	R&D Systems	Cat# MAB6689; RRID:AB_10890940
Rabbit anti-mouse PDGFRβ	Cell Signaling	Cat# 3169S; RRID:AB_2920526
Rabbit anti-mouse Runx1,2,3	Abcam	Cat# AB92336; RRID:AB_2049267
Goat anti-rat AF594	Invitrogen	Cat# A-11007; RRID:AB_10561522
Goat anti-rabbit AF594	Life Technologies	Cat# A11012; RRID:AB_2534079
Goat anti Rabbit AF647	Invitrogen	Cat# A21245; RRID:AB_2535813
Goat anti-rabbit AF488	Invitrogen	Cat# A11008; RRID:AB_143165
Mouse anti-mouse aSMA-Cy3	Sigma	Cat# C6198; RRID:AB_476856
streptavidin AF647	Life Technologies	Cat# S21374; RRID:AB_2336066
DAPI	Thermofisher Scientific	Cat# D1306; RRID:AB_2629482
Rabbit Polyclonal NG2 AF488	Millipore	Cat# ab5320a4; RRID:AB_11203143
Rat anti-mouse PDGFRβ APC	eBioscience	Cat# 17-1402-82; RRID:AB_1548743
Rat anti-mouse PDGFRβ PE	eBioscience	Cat# 136006; RRID:AB_1953271
Rat anti-mouse CD31 PECy7	eBioscience	Cat# 25-0311-82; RRID:AB_271694
Rat anti-mouse CD45 PerCpCy5.5	BD Pharmingen	Cat# 550994; RRID:AB_394003
Rat anti-mouse cKit APC-eFluor780	eBioscience	Cat# 47-1171-82; RRID:AB_1272177
Rat anti-mouse cKit BV421	BD Horizon	Cat# 562609; RRID:AB_11154585
Rat anti-mouse CD41 eFluor450	eBioscience	Cat# 48-0411-82; RRID:AB_1582238
Rat anti-mouse cKit APC	BioLegend	Cat# 105812; RRID:AB_313221
Rat anti-mouse Sca-1 APC-Cy7	BioLegend	Cat# 108126; RRID:AB_10645327
Rat anti-mouse CD4 biotin	BD Biosciences	Cat# 553648; RRID:AB_394968
Rat anti-mouse CD5 biotin	BD Biosciences	Cat# 553018; RRID:AB_394556
Rat anti-mouse CD8a biotin	BD Biosciences	Cat# 553028; RRID:AB_394566
Rat anti-mouse CD11b/Mac-1 biotin	BD Biosciences	Cat# 553309; RRID:AB_394773
Rat anti-mouse CD45R/B220 biotin	BD Biosciences	Cat# 553086; RRID:AB_394616
Rat anti-mouse Gr-1/Ly-6G/C biotin	BD Biosciences	Cat# 553125; RRID:AB_394641
Rat anti-mouse Ter119 biotin	BD Biosciences	Cat# 553672; RRID:AB_394985
Streptavidin PerCp	BioLegend	Cat# 405213; RRID:AB_2920531
Mouse anti-mouse CD45.1-FITC	BioLegend	Cat# 110706; RRID:AB_313495
Mouse anti-mouse CD45.2-Pacific Blue	BioLegend	Cat# 109820; RRID:AB_492872
Rat anti-mouse CD4 PE	Biolegend	Cat# 130310; RRID:AB_2075573
Rat anti-mouse CD8a PE	Biolegend	Cat# 100708; RRID:AB_312747
Rat anti-mouse CD11b APC	Biolegend	Cat# 101212; RRID:AB_312795
Rat anti-mouse CD19 APC-Cy7	Biolegend	Cat# 115530; RRID:AB_830707
Rat anti-mouse Gr1 PE-Cy7	Biolegend	Cat# 108416; RRID:AB_313381
Rat anti-mouse CD146 V450	BD Pharmingen	Cat# 562232; RRID:AB_11152959
Rat anti-mouse CD45 PerCp-Cy5.5	BD Pharmingen	Cat# 550994; RRID:AB_394003
Rat anti-mouse Ter119 APC	BioLegend	Cat# 116212; RRID:AB_313713
Mouse anti-mouse αSMA-FITC	Sigma	Cat# F3777; RRID:AB_476977

**Chemicals, peptides, and recombinant proteins**		

tamoxifen	Sigma	Cat# T5648
corn oil	Sigma	Cat#C8267
Sytox AAD	Thermofisher	Cat# S10349
Dulbecco’s PBS	Sigma	Cat# D8537
fetal calf serum	Life Tech	Cat# 10270106
penicillin/streptomycin	Invitrogen	Cat# 15140–122
collagenase type I	Sigma	Cat# C0130
ammonium chloride solution	Stem Cell Technologies	Cat# 07850
methylcellulose	Stem Cell Technologies Inc.	Cat# M3434
paraformaldehyde	VWR International	Cat# J61899
protein blocking solution	Spring Bioscience	Cat# DPB-125
Fluoromount-G	Southern Biotech	Cat# 0100–01
MyeloCult	Stem Cell Technology	Cat# 05350
αMEM	Gibco	Cat# 22571–020
Glutamax 100X	Gibco	Cat# 35050–038
β-mercaptoethanol	Gibco	Cat# 31350–010
dimethyl sulphoxide	VWR Chemicals	Cat# 23500.260
Triton X-100	Fischer Scientific	Cat# 10591461
BMP4	RD	Cat# 5020-BP/CF
Dexamethasone	Sigma	Cat# D4902
L-ascorbic acid-2-phosphate	Sigma	Cat# A8960
β-Glycerophosphate disodium pentahydrate	Calbiochem	Cat# 35675
DMEM with glutamax	Gibco	Cat# 61965026
alizarin red S	Sigma	Cat# A5533-25G
Nuclease-free water	Ambion	Cat# AM9930

**Critical commercial assays**		

biotin/avidin blocking kit	ThermoFisher Scientific	Cat# 004303

**Deposited data**		

Single-cell RNA seq	This paper	Accession number: GSE162103
Bulk RNA seq	This paper	Accession number: GSE144837

**Experimental models: Cell lines**		

PDGFRβ KO AGM MSCs	This paper	N/A

**Experimental models: Organisms/strains**		

Mouse: PDGFRβ KO	Christer Betsholtz	Soriano (1994)
PDGFRβ-Cre mice	The Jackson Laboratory	(Foo et al., 2006) http://www.informatics.jax.org/allele/MGI:3692784
Rosa26-TdTomato	The Jackson Laboratory	(Madisen et al., 2010) www.jax.org/strain/007914
Rosa26-mTmG mice	The Jackson Laboratory	(Muzumdar et al., 2007) www.jax.org/strain/007576
Runx1-IRES-GFP mice	James Downing	(Lorsbach et al., 2004)
PDGFRβ-P2A-CreER	Henar Cuervo	(Cuervo et al., 2017)
C57BL/6J	Inbred	N/A
Ly5.1 mice	Inbred	N/A

**Software and algorithms**		

GraphPad Prism (v7-9)	GraphPad	www.graphpad.com/scientific-software/prism/
FlowJo Version 10.7.2	Tree Star, Inc	www.flowjo.com/solutions/flowjo/downloads/previous-versions
BD FACSDiva™ (v. 8,and v. 8.0.1, BD Biosciences)	BD Biosciences	www.bdbiosciences.com/en-us
Zen Lite (v2.6) and Pro (v2.3) softwares	Zeiss	www.zeiss.com/microscopy/int/products/microscope-software/zen.html
Fiji/ImageJ software (1.52p and 1.53f51)	FiJi	https://imagej.net/software/fiji/
TopHat2 (v 2.0.10)	(Kim et al., 2013)	https://ccb.jhu.edu/software/tophat/index.shtml
Cufflinks (v 2.1.1)	(Trapnell et al., 2012)	http://cole-trapnell-lab.github.io/cufflinks/
GenomicAlignments (v 1.18.1)	(Lawrence et al., 2013)	https://bioconductor.org/packages/release/bioc/html/GenomicAlignments.html
Cell Ranger (v 3.1.0)	10x Genomics	https://support.10xgenomics.com/single-cell-gene-expression/software/downloads/latest
DropletUtils (v 1.14)	(Lun et al., 2019)	https://bioconductor.org/packages/release/bioc/html/DropletUtils.html
scater (v 1.14.6)	(McCarthy et al., 2017)	https://bioconductor.org/packages/release/bioc/html/scater.html
batchelor (v 1.2.4)	(Haghverdi et al., 2018)	https://bioconductor.org/packages/release/bioc/html/batchelor.html
scran (v 1.22)	(Lun et al., 2016)	https://bioconductor.org/packages/release/bioc/html/scran.html
nichenetr (v 1.1.0)	(Browaeys et al., 2020)	https://github.com/saeyslab/nichenetr
pheatmap (v 1.0.12)	CRAN	https://cran.r-project.org/web/packages/pheatmap/
PANTHER	(Mi et al., 2013)	http://www.pantherdb.org
AmiGO (v 2.5.13)	(Carbon et al., 2009)	http://amigo.geneontology.org/amigo

### Resource availability

#### Lead contact

Further information and requests for resources and reagents should be directed to and will be fulfilled by the Lead Contact, Mihaela Crisan (Mihaela.crisan@ed.ac.uk).

#### Materials availability

This study did not generate new unique reagents.

### Experimental model and subject details

#### Animals and embryo generation

Mice were bred and housed at animal facilities at the the University of Edinburgh, UK and at the University of Illinois at Chicago, USA, in compliance with the local regulations. *PDGFRβ* KO mice (Soriano, 1994) and *PDGFRβ-Cre* (Tg(Pdgfrb-cre)1Rha) mice (Foo et al., 2006) were maintained as heterozygotes on a C57BL/6J background. *Rosa26-TdTomato* mice (Gt(ROSA)26Sor^tm14(CAG-tdTomato)Hze^) (Madisen et al., 2010), *Rosa26-mTmG* mice (Gt(ROSA)26Sor^tm4(ACTB-tdTomato,−EGFP)Luo^/J) (Muzumdar et al., 2007), *Runx1-IRES-GFP* mice (Lorsbach et al., 2004) kindly provided by James Downing (St Jude Children’s Hospital, Memphis, USA) and *PDGFRβ-P2A-CreER* (Cuervo et al., 2017) were maintained as homozygotes on a C57BL/6J background. All experiments were performed under a Project Licence granted by the Home Office (UK), approved by the University of Edinburgh Ethical Review Committee (70–8568/24-04-2017 and PP8962771/23-10-2020) or the Animal Care Committee (ACC) at the University of Illinois at Chicago and conducted in accordance to local guidelines. Adult C57BL/6J WT mice (Ly5.2) and Ly5.1 homozygous and heterozygous (inbred) mice were provided by our animal facilities. All animals used were males and females mixed, from 2 to 4 months old. For experiments involving a time course activation of Cre-recombinase, 1mg of tamoxifen (Sigma) dissolved in corn oil (Sigma), was administered to the pregnant females in a single dose at E7.5, E8.5 or E9.5 while the analysis of the dorsal aorta was performed at E11.5. The day of vaginal plug detection was designated as embryonic day (E) 0.5. Tissues were harvested in a buffer solution (Dulbecco’s PBS, Sigma, D8537) enriched with 10% fetal calf serum (FCS, Life Tech, 10270106) and 1% penicillin/streptomycin (PS, Invitrogen, 15140-122), referred to as PBS/FCS/PS herein, and then kept on ice.

### Method details

#### *In vitro* colony forming unit-culture assays (CFU-C)

Hematopoietic organs (except the FL and adult BM) were dissected and dissociated into single cells in PBS supplemented with 10%FCS and 1%PS (PBS/FCS/PS) and collagenase type I (Sigma, C0130, 0.125% v/v). FL cells were dissociated by pipetting, and BM cells were flushed from both femurs and tibias. BM erythrocytes were lysed with an ammonium chloride solution (Stem Cell Technologies 07850) for 12 min at room temperature (RT). Cells were plated at different densities in methylcellulose (Methocult GF M3434, Stem Cell Technologies Inc.) supplemented with 1%PS, in 35mm petri dishes (Falcon 1008), and incubated at 37°C (5% CO_2_) for 10–12 days. Hematopoietic colonies were counted and distinguished by morphology under an inverted microscope. For co-culture experiments, cells were trypsinized, counted, and analyzed by flow cytometry at day 7. Cells from each well were then plated in methylcellulose medium for 10 to 12 days at 37°C. CFU-C types are defined as: blast forming unit erythroid progenitors (BFU-E), colony forming unit-granulocyte progenitors (CFU-G), macrophage progenitors (CFU-M), granulocyte and macrophage progenitors (CFU-GM), and the most immature granulocyte, erythrocyte, monocyte and megakaryocyte progenitors (CFU-GEMM). Colony numbers obtained were calculated per organ (for all tissues) and in addition, as frequencies per 10^5^ and 10^4^ cells (for E14 FL and adult BM respectively), or per 5 × 10^4^ CD45^+^ cells (for co-culture) and data was analyzed in GraphPad Prism.

#### *In vivo* transplantation assays

Single cell suspensions were injected intravenously into Ly5.1 heterozygous mice (CD45.1^+^ CD45.2^+^) irradiated with a 9Gy (for E14 FL and adult BM) and 9.6Gy (for E11 AGM) split dose. A number of 2 × 10^4^ BM cells (for E11 AGM) or 2 × 10^5^ spleen cells (for E14 FL and adult BM) from Ly5.1 homozygous mice (CD45.1^+^CD45.2^-^) were co-injected. For E14 FL and adult BM transplantations, total BM cells from both femurs and tibias of a positively reconstituted primary recipient with relatively high percentage of donor chimerism were injected into 2 irradiated Ly5.1 heterozygous secondary recipients. Peripheral blood was analyzed by flow cytometry at 1 and 4 months post-transplantation. Mice with ≥5% CD45.1^−^CD45.2^+^ donor cell chimerism were considered positively reconstituted.

#### Three-dimensional wholemount immunostaining

Wholemount immunostainings were performed as previously described (Yokomizo et al., 2012). Briefly, embryos were stained with unconjugated cKit (1:500; BD Bioscience; 553352) or unconjugated NG2 (1:500; Millipore; ab5320) together with biotinylated CD31 (1:500 BD Pharmingen; 553371). The secondary antibody Alexa 647 (1:2500; Invitrogen; A21245) was used to detect cKit or NG2, and streptavidin Cy3 (1:5000; Sigma; S6402) to detect CD31. Embryos were imaged using a Leica SP8 confocal microscope. To count IAHCs, confocal images were analyzed with FIJI/ImageJ using a script. Briefly, a maximum intensity projection of a confocal stack containing the DA was produced and the aorta was manually selected. Intra-aortic ckit^+^ hematopoietic cell clusters were automatically segmented within this area, counted and their areas measured.

#### Immunohistochemistry

Embryos were dissected, and a piece of tissue taken for genotyping. Embryos with their respective placenta and yolk sac were next fixed in 2% paraformaldehyde (PFA) for 20–30min on ice, and washed three times for 10 min with PBS. They were then embeded in 20% sucrose/PBS and incubated overnight (O/N) at 4°C. The following day, embryos were embedded in optimum cutting medium (OCT) and frozen in 100% ethanol cold vapors in dry ice. Sections 7–10μm thick were cut using a cryostat. Slides were post-fixed with 100% cold methanol or 1:1 volume of cold methanol-acetone for 5–7 min at RT, air-dried for another 5min then washed three times 5 min with PBS. Sections to be stained with biotinylated antibodies were blocked with a biotin/avidin blocking kit (ThermoFisher Scientific; 004303). Sections were incubated with an avidin solution for 15min at RT, washed with PBS for 5 min, incubated with a biotin solution for 15 at RT, and washed three times 5min with PBS. All sections were next blocked with a ready-to-use protein blocking solution (Spring Bioscience; DPB-125) or 5% goat serum (Sigma) in PBS in a humidified container for 30 min or 1 h at RT. Immediately after blocking, sections were incubated overnight at 4°C with the directly conjugated primary antibody CD146-AF488 (Rat anti-mouse, Biolegend, 1:100) or with the unconjugated ones: NG2 (Rabbit polyclonal; Millipore; ab5320; 1:100), NG2 (Rat anti-mouse; R&D Systems; MAB6689; 1:100), PDGFRβ (Rabbit anti-mouse; Cell Signaling; 3169S; 1:250), or Runx1,2,3 (Rabbit anti-mouse; Abcam; AB92336; 1:100). The next day, slides were washed three times with PBS and, when required, a secondary antibody was added for 1h at RT in a dark, humidified chamber on sections treated with unconjugated antibodies. The secondary antibodies used were AF594 (Goat anti-rat; Invitrogen; A-11007; 1:500), AF594 (Goat anti-rabbit; Life Technologies; A11012; 1:500), AF647 (Goat anti Rabbit, Invitrogen, A21245, 1:300), AF488 (Goat anti-rabbit; Invitrogen; A11008; 1:500). For NG2 and PDGFRβ double-staining, antibodies were incubated separately in two consecutive overnights at 4°C. For Runx1,2,3 antibody staining, 0.5% Triton X-100 (Acros) was added to all solutions. Following secondary antibody staining, slides were washed with PBS, and biotinylated CD31 (BD Pharmingen; 553371; 1:50) and/or directly conjugated aSMA-Cy3 (Sigma, C6198, 1:100) were added for 2h at RT. After washing, sections stained with CD31 were additionally incubated with streptavidin-Cy3 (Sigma; S6402-1mL; 1:250) or streptavidin AF647 (Life Technologies; S21374; 1:500) for 30 min RT and washed with PBS. All sections were stained with DAPI (1:500; Thermofisher Scientific, D1306) for 15 min at RT, washed, and mounted using Fluoromount-G (Southern Biotech, 0100-01). Stained sections were imaged using an inverted widefield fluorescence microscope (Zeiss Observer) or a Leica SP8 confocal microscope, deconvolved using Huygens Professional (version 19.04) deconvolution wizard, and tiled images (taken with a 63x oil objective) were further stitched either in simultaneous with Huygens deconvolution, or with FIJI/ImageJ after deconvolution. Pictures were analyzed with Fiji/ImageJ software. Maximum intensity projections were made from 1 to 10 z-stacks using FIJI/ImageJ.

#### Flow cytometry

Single cell suspensions were stained with various combinations of antibodies containing NG2 AF488 (1:100; Millipore; ab5320a4), PDGFRβ APC (1:250; eBioscience; 17-1402-82), PDGFRβ PE (1:250; eBioscience; 136006), CD31 PECy7 (1:4000; eBioscience; 25-0311-82), CD45 PerCpCy5.5 (1:400; BD Pharmingen; 550994), cKit APC-eFluor780 (1:800; eBioscience; 47-1171-82), cKit BV421 (1:500; BD Horizon; 562609), and CD41 eFluor450 (1:100; eBioscience; 48-0411-82). For LSK analysis, BM cells were harvested and red blood cells lysed as described above. BM cells were initially stained with a cocktail of antibodies containing hematopoietic lineage biotinylated antibodies (Lin), cKit APC (BioLegend; 105812; 1:200) and Sca-1 APC-Cy7 (BioLegend; 122514; 1:200) diluted in BM FACS buffer (PBS/2%FCS/1%PS/2mM EDTA) and incubated for 30min at 4°C. Lin antibodies used are all from BD Biosciences: CD4 (553648; 1:1600), CD8 (553018; 1:800), CD8a (553028; 1:800), CD11b/Mac-1 (553309; 1:200), CD45R/B220 (553086; 1:200), Gr-1/Ly-6G/C (553125; 1:100), Ter119 (553672; 1:50). Cells were next washed and incubated for 30 min at 4°C with Streptavidin PerCp (BioLegend; 405213; 1:200). For peripheral blood analyses, red cells were lysed as described above, and cells were stained for 30min at 4°C with CD45.1-FITC (1:1000; BioLegend; 110706) and CD45.2-Pacific Blue (1:1000; BioLegend; 109802), CD4 PE (1:5000; Biolegend; 130310), CD8a PE (1:500; Biolegend; 100708), CD11b APC (1:1000; Biolegend; 101212), CD19 APC-Cy7 (1:1000; Biolegend; 115530) and Gr1 PE-Cy7 (1:2000; Biolegend; 108416). Phenotypic characterization of MSCs was performed by flow cytometry at passage 3 (P3) and P6 of culture. MSCs were trypsinized with 0.25% Trypsin + EDTA (Life Tech; 25200-072), washed with PBS/FCS/PS and counted. Cells were incubated for 30 min and 4°C in the dark with an antibody cocktail containing PDGFRβ PE (1:100; eBioscience; 136006), CD31 PE-Cy7 (1:4000; eBioscience, 25-0311-82), CD146 V450 (1:200, BD Pharmingen, 562232) and CD45 (PerCp-Cy5.5; 1:400; BD Pharmingen; 550994). Cells were then washed and resuspended in 300μL of the PBS/FCS/PS. Unstained samples and full minus one (FMO) tubes were used as negative controls. Sytox AAD (1:1000, Thermofisher, S10349) was used as a dead/live-cell dye. For co-culture experiments, cells were trypsinized and counted, before a fraction of 100μL from each sample was analyzed for the presence of CD45^+^ cells (CD45 antibody, PerCp-Cy5.5; 1:400; BD Pharmingen; 550994) by flow cytometry. Erythroid cells were stained with antibody against Ter119 (APC, 1:200, BioLegend, 116212). All analyses were performed on a BD LSR Fortessa 4 laser (BD Biosciences) and the software BD FACSDiva™ and FlowJo.

#### Fluorescence-activated cell sorting (FACS)

Single cell suspensions of E10 and E11 C57BL/6J mouse AGMs were sorted based on PDGFRβ^+/−^ expression while PDGFRβ-Cre; R26TdTomato AGM, based on tdTomato^+/−^ expression. As all functional E11 HSPCs have been reported cKit^+^ (Crisan et al., 2016), E11 cells were also stained with cKit for further enrichment. Sorted cells were seeded in methylcellulose or injected into irradiated recipient mice as described above. For co-culture experiments, BM cells from C57BL/6J adult mice were harvested from both femurs and tibias, and processed and stained as described above for analysis. For co-culture with embryonic cells, nine E11 AGM harvested from C57BL/6J mouse embryos were pooled and endothelial cells and hematopoietic progenitors were sorted based on CD31^+^CD45^−^ckit^-^ and CD31^+^ckit^+^, respectively. For bulk RNA sequencing, CD31^−^CD45^−^ckit^-^ DP (NG2^+^PDGFRβ^+^), PDGFRβ-S (NG2^−^PDGFRβ^+^) and DN (NG2^−^PDGFRβ^-^) sorted cells were directly collected into lysis buffer (see scRNA-seq method for details). For Cre genotyping, seven E11 C57BL/6J mouse AGMs were pooled and sorted for CD31^+^PDGFRβ^−^ and CD31^−^PDGFRβ^+^. DAPI (1:1000, Thermofisher Scientific, D1306) or Hoechst 33,258 (1:10,000, Molecular Probes) was used as a live/dead cell dye. All antibodies listed here are detailed in the flow cytometry protocol, above. Cell sorting was done using BD FACS ARIA III SORP 4 laser or FACS Aria Fusion cell sorter (BD Biosciences) and the software BD FACSDiva™ (v. 8 and v. 8.0.1, BD Biosciences).

#### Mesenchymal stem/stromal cell (MSC) line derivation and culture

Single AGMs were dissected and incubated with collagenase type I (1:20, Sigma, C0130) in a water bath at 37°C for 45 min. A piece of tissue (YS or head) from each embryo was used for genotyping. Following digestion, AGMs were mechanically dissociated and washed with PBS/FCS/PS then centrifuged for 10 min, at 2000 rpm and 4°C. Cells obtained from one AGM were seeded in one well in a 6-well plate pre-coated with 0.1% gelatin (Sigma; G1890) (= passage 0). Each well was filled with 3 mL of MSC media, consisting of 50% MyeloCult (Stem Cell Technology M5300; 05350), 15% FCS, 35% αMEM (Gibco; 22571-020), 1% PS, 1% Glutamax (100x; Gibco; 35050-038) and 0,02% β-mercaptoethanol (Gibco; 31350-010), previously described (Oostendorp et al., 2002a, 2002). The medium was changed weekly. When cells reached >90% confluence (approximately 1 week), they were passaged from 6-well plates to T75 flasks pre-coated with 0.1% gelatin (= passage 1) and approximately 1 week later, expanded to 3 T75 flasks (≥ passage 2) which were maintained for more passages in the incubator at 37°C and 5% CO_2_ at a ratio of 1:3 before being frozen. Around one million cells were split per cryovial with 90% cold FCS and 10% dimethyl sulphoxide (DMSO, VWR Chemicals; 23500.260) in liquid nitrogen for further analysis.

#### Immunocytochemistry

A number of 40,000 cells were seeded in a 24-well plate pre-coated with 0.1% gelatin and 500μL of MSC media and kept at 37°C in the incubator for 24 h. When cells were confluent, the media was carefully removed and cells were washed with PBS and fixed with 4% paraformaldehyde (PFA, VWR International, J61899) for 10 min at 4°C than washed twice for 10 min with PBS. Wells were next incubated with Triton 0.5% (Triton X-100, Fischer Scientific, 10591461) in PBS for 10 min and washed twice for 10 min with PBS. All samples were incubated with a ready-to-use protein blocking solution (Spring Bioscience, DPB-125) for 15 min at RT. Followed by a washing step with PBS and a second blocking with 10% goat serum for 1h at RT. αSMA-FITC anti-mouse antibody (Sigma, F3777, 1:100) was incubated for 2h at RT in the dark. Negative controls were incubated with blocking serum. Wells were then washed three times with PBS for 10 min each and stained with DAPI (1:500, Thermofisher Scientific, D1306) for 15 min at RT in the dark. Finally, cells were washed with PBS and imaged using an inverted widefield fluorescence microscope (Axio Observer, Zeiss). Pictures were acquired and analyzed with Zen and Fiji/ImageJ software.

#### Co-culture experiments

A number of 1.5 × 10^5^ or 7.5 × 10^4^ P4-P6 MSCs from each genotype were seeded per well in 6-well or 12-well plates pre-coated with 0.1% gelatin in MSC media and kept in the incubator at 37°C, 5% CO_2_. At 90% confluence (day 4), MSCs were irradiated at 30 Gy using a Caesium-137 Gammacell 40 Exactor irradiator and the medium was refreshed. The same day, 3000 and 500 sorted WT BM-derived LSK cells were added to each well in 6-well and 12-well plates, respectively. Irradiated MSCs alone were used as control. For co-culture with WT AGM ECs or HSPCs, 7.5 × 10^4^ P6-P11 MSCs from each genotype were seeded per well in 12-well plates pre-coated with 0.1% gelatin in MSC media and kept in the incubator at 37°C, 5% CO_2_. At 90% confluence (day 4), MSCs were irradiated as described above. The same day, 1 embryo equivalent of HSPCs or 500 ECs sorted from a pool of WT AGMs were added to each well. For rescue experiments, BMP4 (RD; 5020-BP/CF) was added to KO AGM MSC co-cultures at 20ng/mL. At day 7, all wells were imaged using Evos Digital Inverted Microscope (AMG) for the co-culture with the BM, and Nikon TSR2 microscope for co-culture with AGM ECs/HPSCs, then cells were trypsinized and counted. A fraction of 100μL from each sample was analyzed for the presence of CD45^+^ cells by flow cytometry (described above). The remaining cells were used for CFU-C assays as described.

#### Osteogenesis assay

Two media were used for the osteogenesis assay: osteogenic and control media. The osteogenic medium consisted of 0, 1% of Dexamethasone (1mM, Sigma, D4902), 1% of L-ascorbic acid-2-phosphate (L-AA, 5mM, Sigma, A8960), 1% of β-Glycerophosphate disodium pentahydrate (β-GP, 1M, Calbiochem; 35675), 10% of FCS, 1% of PS and 87% of DMEM with glutamax (Gibco, 61965026). The control medium contained 89% of DMEM with glutamax (Gibco, 61965026), 10% of FCS, and 1% PS. For osteogenic differentiation, 4 × 10^4^ MSCs from each genotype were seeded per well in a 24-well plate. After 24h of culture, the MSC medium was replaced with 500μL of either osteogenic or control media (= day 0 of differentiation). The samples were incubated at 37°C and 5% CO_2_ for 21 days. The media were refreshed three times per week. To visualise calcium deposition, we prepared an alizarin red solution with 2g of alizarin red S (Sigma, A5533-25G) diluted in 100 mL of Milli-Q water. The solution was next filtered and the pH adjusted to 4.1–4.3 and then stored in the dark. The control and osteogenic media were discarded from each well and washed with Milli-Q water. The samples were fixed with 4% PFA for 10 min and washed again. A volume of 500μL of the alizarin red solution was added for 15 min, followed by two washes with Milli-Q water. Lastly, full wells were imaged with a brightfield microscope (Axio Observer, Zeiss) and pictures acquired and analyzed with Zen and Fiji/ImageJ software.

#### Bulk RNA sequencing and analysis

Cells were sorted and collected directly into 20μL of lysis buffer containing Nuclease-free water (Ambion AM9930), 0.2% Triton and 1/20 RNAse inhibitor. We generated full-length cDNA from 3.4 μL of cell lysate according to the Smarter2 technology as described (Picelli et al., 2013). 500 pg of cDNA was tagmented with Illumina’s Nextera XT sample prep kit (Illumina Inc., U.S.A) and converted into sequencing libraries. After pooling, single-read 43bp sequencing was performed on Illumina HiSeq2000 using the Truseq v3 sequencing chemistry (Illumina Inc., U.S.A). TopHat2 version 2.0.10 (Kim et al., 2013) was used to align the reads to the mouse reference genome (mm10). Gene expression values for RefSeq transcripts were calculated as TPMs using Cufflinks (Trapnell et al., 2012) (2.1.1). Raw reads were counted with the summarizeOverlaps function with the GENCODE M19 gene annotation using the union mode from the Bioconductor Genomic Alignments package (Lawrence et al., 2013) (v1.18.1).

#### Single cell RNA sequencing and data analysis

##### Library preparation and sequencing

Cells were processed through the 10x Genomics Chromium Single Cell Platform using the Single Cell 3′ Library and Gel Bead Kit v3 (10X Genomics) as per the manufacturer’s instructions. In brief, AGMs from PDGRFβ^+/+^ and PDGRFβ^−/−^ embryos were dissociated (as described above), washed, counted and assessed for viability using a Bio-Rad TC20. Cells were then loaded at a concentration for the recovery of 7000 cells and processed through the 10x Chromium Controller. Single cell libraries were obtained according to the manufacturer’s protocol. RNA concentration was obtained using Qubit RNA HS (Thermo-Fisher) and the quality of the libraries was verified using the LabChip GX24 (PerkinElmer). Libraries were then sent for sequencing to Edinburgh Genomics.

##### Data analysis

Alignment of scRNA-seq data and barcode counting was performed using 10X Genomics Cell Ranger (v3.1.0) with reference dataset mm10/GRCm38–3.0.0. Unfiltered UMI count matrices from Cell Ranger were used as input for downstream analysis following the OSCA Bioconductor workflow (Amezquita et al., 2020). The EmptyDrops method (Lun et al., 2019) was used to remove cells predicted to contain only ambient RNA, calling 11,037 (WT) and 15,848 (KO) cells at the default false discovery rate (FDR) of 0.1%. Quality metrics (library size, number of expressed features and percentage of mitochondrial reads) were computed for the remaining cells using the scater Bioconductor package (McCarthy et al., 2017) (v1.14.6). Using the median absolute deviation (MAD) definition of outliers, we removed cells with any quality metric more extreme than 3 MADs from the median. 860 cells with detected PDGFRβ expression were removed from the KO sample. After these quality filtering steps, 10,091 (WT) and 14,577 (KO) cells remained for downstream analysis. For both WT and KO samples, the filtered UMI count matrixes were log-normalized, applying the deconvolution method of Lun et al. to compute size factors for all cells (Lun et al., 2016). Technical noise was modeled using a Poisson-based trend, serving as a lower bound for the variance of endogenous genes. Dimensionality reduction for denoising was performed with principal component analysis (PCA) using the modeled trend. Shared nearest neighbor (SNN) graphs were constructed using the PCA-reduced dataset and used as input to the Walktrap community finding algorithm to obtain cell clusters. These clusters were used as an initial approximation to known cell types; this approximation was refined based on the distribution and expression levels of literature-derived marker genes. Doublet scores for each cell were computed by *in silico* simulation of putative doublet expression profiles using the scran Bioconductor package. No cell clusters comprising only cells with high doublet scores were observed; we therefore kept all cells, retaining their doublet scores to inform downstream analysis. A fast version of the mutual nearest neighbors (MNN) method from the batchelor Bioconductor package (Haghverdi et al., 2018) (v1.2.4) was used to integrate WT and KO data. Projection of all cells into a shared t-distributed stochastic neighbor embedding (t-SNE) space confirmed consistency of cell type annotations. Differential expression between WT and KO cells was computed by Wilcoxon rank-sum test between appropriate groups, using the pairwiseWilcox function from the scran Bioconductor package. Genes with FDR<0.05 were considered to be significantly differentially expressed. Heatmaps were generated with the pheatmap Bioconductor package (v1.0.12), depicting either median expression of cell populations, or expression of single cells, as appropriate. Log normalized expression data was used for visualing cells and populations within a single sample; for cross-sample comparisons, batch-corrected expression data generated by MNN integration was used. Enrichment analysis with the PANTHER classification system (Mi et al., 2013) was used to identify Gene Ontology (GO) biological processes overrepresented among significantly differentially expressed genes. Fold enrichment of GO biological processes was computed by Fisher’s Exact Test; processes with FDR<0.05 were considered to be significantly enriched. Additional data mining of GO terms was performed using AmiGO (Carbon et al., 2009) (v.2.5.13). Ligand-receptor interactions were predicted using the nichenetr (v.1.1.0) R package (Browaeys et al., 2020). We considered genes to be expressed in a given cell type if they had detectable expression in at least 25% of cells of that cell type. The top 10 ligands expressed in DP cells were chosen based on Pearson correlation coefficient; target genes expressed in ECs and IAHCs were inferred using these ligands. Ligand-receptor network inference was used to predict and score potential interactions.

### Quantification and statistical analysis

All graphs and statistical analyses were done with GraphPad Prism. We performed Shapiro-Wilk tests to assess the normality of the distribution and determine the usage of parametric or non-parametric statistical tests. Most CFU-Cs were analyzed either with one way ANOVA followed by a Tukey post-hoc test, or a Kruskal-Wallis test, followed by a Dunn’s post-hoc test (all details can be found in the supplementary tables). CFU-Cs with only 2 conditions and all flow cytometry data were analyzed using unpaired t-tests or Mann-Whitney tests, depending on the normality of the distribution. Fisher’s Exact test was used to analyze the differences in the percentage of single live cells between WT and KO populations obtained by scRNA-seq and Wilcoxon rank-sum test was used to compare the gene expression. A Z test for proportions was used when determining the number of MSC lines with osteogenic potential. Error bars represent standard deviations (SD) and the significance was determined for p < 0.05 and described in the figure legends. ‘N’ indicates the number of experiments, and ‘n’ indicates the number of biological replicates.

## Data Availability

• The single-cell RNA-seq and the bulk RNA-seq data from this study have been deposited in GEO database (https://www.ncbi.nlm.nih.gov/geo/) and are publicly available as of the date of publication. Accession numbers are listed in the key resources table. Microscopy data reported in this paper will be shared by the lead contact upon request. • No original code had been generated for this paper. • Any additional information required to reanalyze the data reported in this paper is available from the lead contact upon request.

## Supplemental information

Document S1. Figures S1–S7 and Tables S1–S7

Document S2. Article plus supplemental information

## References

[bib1] Amezquita R.A., Lun A.T.L., Becht E., Carey V.J., Carpp L.N., Geistlinger L., Marini F., Rue-Albrecht K., Risso D., Soneson C. (2020). Orchestrating single-cell analysis with Bioconductor. Nat. Methods.

[bib2] Armulik A., Abramsson A., Betsholtz C. (2005). Endothelial/pericyte interactions. Circ. Res..

[bib3] Azzoni E., Frontera V., McGrath K.E., Harman J., Carrelha J., Nerlov C., Palis J., Jacobsen S.E.W., de Bruijn M.F. (2018). Kit ligand has a critical role in mouse yolk sac and aorta-gonad-mesonephros hematopoiesis. EMBO Rep..

[bib4] Barrionuevo F., Taketo M.M., Scherer G., Kispert A. (2006). Sox9 is required for notochord maintenance in mice. Dev. Biol..

[bib5] Bertrand J.Y., Cisson J.L., Stachura D.L., Traver D. (2010). Notch signaling distinguishes 2 waves of definitive hematopoiesis in the zebrafish embryo. Blood.

[bib6] Bhatia M., Bonnet D., Wu D., Murdoch B., Wrana J., Gallacher L., Dick J.E. (1999). Bone morphogenetic proteins regulate the developmental program of human hematopoietic stem cells. J. Exp. Med..

[bib7] Bigas A., Espinosa L. (2012). Hematopoietic stem cells: to be or Notch to be. Blood.

[bib8] Boisset J.C., van Cappellen W., Andrieu-Soler C., Galjart N., Dzierzak E., Robin C. (2010). In vivo imaging of haematopoietic cells emerging from the mouse aortic endothelium. Nature.

[bib9] Browaeys R., Saelens W., Saeys Y. (2020). NicheNet: modeling intercellular communication by linking ligands to target genes. Nat. Methods.

[bib10] Burns C.E., Traver D., Mayhall E., Shepard J.L., Zon L.I. (2005). Hematopoietic stem cell fate is established by the Notch-Runx pathway. Genes Dev..

[bib11] Caplan A.I., Correa D. (2011). PDGF in bone formation and regeneration: new insights into a novel mechanism involving MSCs. J. Orthop. Res..

[bib12] Carbon S., Ireland A., Mungall C.J., Shu S., Marshall B., Lewis S., Ami G.O.H., Web Presence Working Group (2009). AmiGO: online access to ontology and annotation data. Bioinformatics.

[bib13] Chadwick K., Wang L., Li L., Menendez P., Murdoch B., Rouleau A., Bhatia M. (2003). Cytokines and BMP-4 promote hematopoietic differentiation of human embryonic stem cells. Blood.

[bib14] Chanda B., Ditadi A., Iscove N.N., Keller G. (2013). Retinoic acid signaling is essential for embryonic hematopoietic stem cell development. Cell.

[bib15] Charbord P., Pouget C., Binder H., Dumont F., Stik G., Levy P., Allain F., Marchal C., Richter J., Uzan B. (2014). A systems biology approach for defining the molecular framework of the hematopoietic stem cell niche. Cell Stem Cell.

[bib16] Chen M.J., Yokomizo T., Zeigler B.M., Dzierzak E., Speck N.A. (2009). Runx1 is required for the endothelial to haematopoietic cell transition but not thereafter. Nature.

[bib17] Cheung M., Briscoe J. (2003). Neural crest development is regulated by the transcription factor Sox9. Development.

[bib18] Ciriza J., Thompson H., Petrosian R., Manilay J.O., Garcia-Ojeda M.E. (2013). The migration of hematopoietic progenitors from the fetal liver to the fetal bone marrow: lessons learned and possible clinical applications. Exp. Hematol..

[bib19] Clements W.K., Kim A.D., Ong K.G., Moore J.C., Lawson N.D., Traver D. (2011). A somitic Wnt16/Notch pathway specifies haematopoietic stem cells. Nature.

[bib20] Corselli M., Chin C.J., Parekh C., Sahaghian A., Wang W., Ge S., Evseenko D., Wang X., Montelatici E., Lazzari L. (2013). Perivascular support of human hematopoietic stem/progenitor cells. Blood.

[bib21] Costa G., Kouskoff V., Lacaud G. (2012). Origin of blood cells and HSC production in the embryo. Trends Immunol..

[bib22] Crisan M., Dzierzak E. (2016). The many faces of hematopoietic stem cell heterogeneity. Development.

[bib23] Crisan M., Yap S., Casteilla L., Chen C.W., Corselli M., Park T.S., Andriolo G., Sun B., Zheng B., Zhang L. (2008). A perivascular origin for mesenchymal stem cells in multiple human organs. Cell Stem Cell.

[bib24] Crisan M., Kartalaei P.S., Vink C.S., Yamada-Inagawa T., Bollerot K., van IJcken W., van der Linden R., de Sousa Lopes S.M.C., Monteiro R., Mummery C., Dzierzak E. (2015). BMP signalling differentially regulates distinct haematopoietic stem cell types. Nat. Commun..

[bib25] Crisan M., Solaimani Kartalaei P., Neagu A., Karkanpouna S., Yamada-Inagawa T., Purini C., Vink C.S., van der Linden R., van Ijcken W., Chuva de Sousa Lopes S. (2016). BMP and Hedgehog regulate distinct AGM hematopoietic stem cells ex vivo. Stem Cell Rep..

[bib26] Crosse E.I., Gordon-Keylock S., Rybtsov S., Binagui-Casas A., Felchle H., Nnadi N.C., Kirschner K., Chandra T., Tamagno S., Webb D.J. (2020). Multi-layered spatial transcriptomics identify secretory factors promoting human hematopoietic stem cell development. Cell Stem Cell.

[bib27] Cuervo H., Pereira B., Nadeem T., Lin M., Lee F., Kitajewski J., Lin C.S. (2017). PDGFR beta-P2A-CreER(T2) mice: a genetic tool to target pericytes in angiogenesis. Angiogenesis.

[bib28] Damm E.W., Clements W.K. (2017). Pdgf signalling guides neural crest contribution to the haematopoietic stem cell specification niche. Nat. Cell Biol..

[bib29] de Bruijn M.F., Ma X., Robin C., Ottersbach K., Sanchez M.J., Dzierzak E. (2002). Hematopoietic stem cells localize to the endothelial cell layer in the midgestation mouse aorta. Immunity.

[bib30] Ding L., Saunders T.L., Enikolopov G., Morrison S.J. (2012). Endothelial and perivascular cells maintain haematopoietic stem cells. Nature.

[bib31] Durand C., Robin C., Dzierzak E. (2006). Mesenchymal lineage potentials of aorta-gonad-mesonephros stromal clones. Haematolagica.

[bib32] Durand C., Robin C., Bollerot K., Baron M.H., Ottersbach K., Dzierzak E. (2007). Embryonic stromal clones reveal developmental regulators of definitive hematopoietic stem cells. Proc. Natl. Acad. Sci. USA.

[bib33] Eilken H.M., Nishikawa S.I., Schroeder T. (2009). Continuous single-cell imaging of blood generation from haemogenic endothelium. Nature.

[bib34] Fadlullah M.Z.H., Neo W.H., Lie-A-Ling M., Thambyrajah R., Patel R., Mevel R., Aksoy I., Do Khoa N., Savatier P., Fontenille L. (2022). Murine AGM single-cell profiling identifies a continuum of hemogenic endothelium differentiation marked by ACE. Blood.

[bib35] Fitch S.R., Kimber G.M., Wilson N.K., Parker A., Mirshekar-Syahkal B., Gottgens B., Medvinsky A., Dzierzak E., Ottersbach K. (2012). Signaling from the sympathetic nervous system regulates hematopoietic stem cell emergence during embryogenesis. Cell Stem Cell.

[bib36] Fitch S.R., Kapeni C., Tsitsopoulou A., Wilson N.K., Gottgens B., de Bruijn M.F., Ottersbach K. (2020). Gata3 targets Runx1 in the embryonic haematopoietic stem cell niche. IUBMB Life.

[bib37] Foo S.S., Turner C.J., Adams S., Compagni A., Aubyn D., Kogata N., Lindblom P., Shani M., Zicha D., Adams R.H. (2006). Ephrin-B2 controls cell motility and adhesion during blood-vessel-wall assembly. Cell.

[bib38] Gao L., Tober J., Gao P., Chen C., Tan K., Speck N.A. (2018). RUNX1 and the endothelial origin of blood. Exp. Hematol..

[bib39] Gekas C., Rhodes K.E., Van Handel B., Chhabra A., Ueno M., Mikkola H.K.A. (2010). Hematopoietic stem cell development in the placenta. Int. J. Dev. Biol..

[bib40] Gering M., Patient R. (2010). Notch signalling and haematopoietic stem cell formation during embryogenesis. J. Cell. Physiol..

[bib41] Goessling W., North T.E., Loewer S., Lord A.M., Lee S., Stoick-Cooper C.L., Weidinger G., Puder M., Daley G.Q., Moon R.T., Zon L.I. (2009). Genetic interaction of PGE2 and Wnt signaling regulates developmental specification of stem cells and regeneration. Cell.

[bib42] Greenbaum A., Hsu Y.M.S., Day R.B., Schuettpelz L.G., Christopher M.J., Borgerding J.N., Nagasawa T., Link D.C. (2013). CXCL12 in early mesenchymal progenitors is required for haematopoietic stem-cell maintenance. Nature.

[bib43] Guimaraes-Camboa N., Cattaneo P., Sun Y., Moore-Morris T., Gu Y., Dalton N.D., Rockenstein E., Masliah E., Peterson K.L., Stallcup W.B. (2017). Pericytes of multiple organs do not behave as mesenchymal stem cells in vivo. Cell Stem Cell.

[bib44] Haghverdi L., Lun A.T.L., Morgan M.D., Marioni J.C. (2018). Batch effects in single-cell RNA-sequencing data are corrected by matching mutual nearest neighbors. Nat. Biotechnol..

[bib45] Hellstrom M., Gerhardt H., Kalen M., Li X.R., Eriksson U., Wolburg H., Betsholtz C. (2001). Lack of pericytes leads to endothelial hyperplasia and abnormal vascular morphogenesis. J. Cell Biol..

[bib46] Hellstrom M., Kalen M., Lindahl P., Abramsson A., Betsholtz C. (1999). Role of PDGF-B and PDGFR-beta in recruitment of vascular smooth muscle cells and pericytes during embryonic blood vessel formation in the mouse. Development.

[bib47] Imanirad P., Kartalaei P.S., Crisan M., Yamada-Inagawa T., van der Linden R., Vink C., Speck N., Dzierzak E. (2012). Role of hypoxia in hematopoietic progenitor and stem cell generation and function during mouse ontogeny. Exp. Hematol..

[bib48] Isern J., Martin-Antonio B., Ghazanfari R., Martin A.M., Lopez J.A., del Toro R., Sanchez-Aguilera A., Arranz L., Martin-Perez D., Suarez-Lledo M. (2013). Self-renewing human bone marrow mesenspheres promote hematopoietic stem cell expansion. Cell Rep..

[bib49] Jaffredo T., Gautier R., Eichmann A., Dieterlen-Lievre F. (1998). Intraaortic hemopoietic cells are derived from endothelial cells during ontogeny. Development.

[bib50] Kaminski W.E., Lindahl P., Lin N.L., Broudy V.C., Crosby J.R., Hellstrom M., Swolin B., Bowen-Pope D.F., Martin P.J., Ross R. (2001). Basis of hematopoietic defects in platelet-derived growth factor (PDGF)-B and PDGF beta-receptor null mice. Blood.

[bib51] Kim D., Pertea G., Trapnell C., Pimentel H., Kelley R., Salzberg S.L. (2013). TopHat2: accurate alignment of transcriptomes in the presence of insertions, deletions and gene fusions. Genome Biol..

[bib52] Kissa K., Herbomel P. (2010). Blood stem cells emerge from aortic endothelium by a novel type of cell transition. Nature.

[bib53] Kranc K.R., Schepers H., Rodrigues N.P., Bamforth S., Villadsen E., Ferry H., Bouriez-Jones T., Sigvardsson M., Bhattacharya S., Jacobsen S.E., Enver T. (2009). Cited2 is an essential regulator of adult hematopoietic stem cells. Cell Stem Cell.

[bib54] Kumano K., Chiba S., Kunisato A., Sata M., Saito T., Nakagami-Yamaguchi E., Yamaguchi T., Masuda S., Shimizu K., Takahashi T. (2003). Notch1 but not Notch2 is essential for generating hematopoietic stem cells from endothelial cells. Immunity.

[bib55] Kunisaki Y., Bruns I., Scheiermann C., Ahmed J., Pinho S., Zhang D., Mizoguchi T., Wei Q., Lucas D., Ito K. (2013). Arteriolar niches maintain haematopoietic stem cell quiescence. Nature.

[bib56] Landry J.R., Kinston S., Knezevic K., Donaldson I.J., Green A.R., Gottgens B. (2005). Fli1, Elf1, and Ets1 regulate the proximal promoter of the LMO2 gene in endothelial cells. Blood.

[bib57] Lawrence M., Huber W., Pages H., Aboyoun P., Carlson M., Gentleman R., Morgan M.T., Carey V.J. (2013). Software for computing and annotating genomic ranges. PLoS Comput. Biol..

[bib58] Li Z., Lan Y., He W., Chen D., Wang J., Zhou F., Wang Y., Sun H., Chen X., Xu C. (2012). Mouse embryonic head as a site for hematopoietic stem cell development. Cell Stem Cell.

[bib59] Li Z., Vink C.S., Mariani S.A., Dzierzak E. (2016). Subregional localization and characterization of Ly6aGFP-expressing hematopoietic cells in the mouse embryonic head. Dev. Biol..

[bib60] Lim S.E., Esain V., Kwan W., Theodore L.N., Cortes M., Frost I.M., Liu S.Y., North T.E. (2017). HIF1 alpha-induced PDGFR beta signaling promotes developmental HSC production via IL-6 activation. Exp. Hematol..

[bib61] Lindahl P., Johansson B.R., Leveen P., Betsholtz C. (1997). Pericyte loss and microaneurysm formation in PDGF-B-deficient mice. Science.

[bib62] Lorsbach R.B., Moore J., Ang S.O., Sun W., Lenny N., Downing J.R. (2004). Role of RUNX1 in adult hematopoiesis: analysis of RUNX1-IRES-GFP knock-in mice reveals differential lineage expression. Blood.

[bib63] L Lun A.T., Bach K., Marioni J.C. (2016). Pooling across cells to normalize single-cell RNA sequencing data with many zero counts. Genome Biol..

[bib64] Lun A.T.L., Riesenfeld S., Andrews T., Dao T.P., Gomes T., Marioni J.C., Participants in the 1st Human Cell Atlas (2019). EmptyDrops: distinguishing cells from empty droplets in droplet-based single-cell RNA sequencing data. Genome Biol..

[bib65] Madisen L., Zwingman T.A., Sunkin S.M., Oh S.W., Zariwala H.A., Gu H., Ng L.L., Palmiter R.D., Hawrylycz M.J., Jones A.R. (2010). A robust and high-throughput Cre reporting and characterization system for the whole mouse brain. Nat. Neurosci..

[bib66] Mariani S.A., Li Z., Rice S., Krieg C., Fragkogianni S., Robinson M., Vink C.S., Pollard J.W., Dzierzak E. (2019). Pro-inflammatory aorta-associated macrophages are involved in embryonic development of hematopoietic stem cells. Immunity.

[bib67] McCarthy D.J., Campbell K.R., Lun A.T.L., Wills Q.F. (2017). Scater: pre-processing, quality control, normalization and visualization of single-cell RNA-seq data in R. Bioinformatics.

[bib68] McGarvey A.C., Rybtsov S., Souilhol C., Tamagno S., Rice R., Hills D., Godwin D., Rice D., Tomlinson S.R., Medvinsky A. (2017). A molecular roadmap of the AGM region reveals BMPER as a novel regulator of HSC maturation. J. Exp. Med..

[bib69] McReynolds L.J., Gupta S., Figueroa M.E., Mullins M.C., Evans T. (2007). Smad1 and Smad5 differentially regulate embryonic hematopoiesis. Blood.

[bib70] Medvinsky A., Dzierzak E. (1996). Definitive hematopoiesis is autonomously initiated by the AGM region. Cell.

[bib71] Mendes S.C., Robin C., Dzierzak E. (2005). Mesenchymal progenitor cells localize within hematopoietic sites throughout ontogeny. Development.

[bib72] Mendez-Ferrer S., Michurina T.V., Ferraro F., Mazloom A.R., Macarthur B.D., Lira S.A., Scadden D.T., Ma'ayan A., Enikolopov G.N., Frenette P.S. (2010). Mesenchymal and haematopoietic stem cells form a unique bone marrow niche. Nature.

[bib73] Mi H.Y., Muruganujan A., Casagrande J.T., Thomas P.D. (2013). Large-scale gene function analysis with the PANTHER classification system. Nat. Protoc..

[bib74] Mikkola H.K.A., Fujiwara Y., Schlaeger T.M., Traver D., Orkin S.H. (2003). Expression of CD41 marks the initiation of definitive hematopoiesis in the mouse embryo. Blood.

[bib75] Moretti A., Caron L., Nakano A., Lam J.T., Bernshausen A., Chen Y., Qyang Y., Bu L., Sasaki M., Martin-Puig S. (2006). Multipotent embryonic isl1+ progenitor cells lead to cardiac, smooth muscle, and endothelial cell diversification. Cell.

[bib76] Muzumdar M.D., Tasic B., Miyamichi K., Li L., Luo L.Q. (2007). A global double-fluorescent cre reporter mouse. Genesis.

[bib77] Nagel S., Pommerenke C., MacLeod R.A.F., Meyer C., Kaufmann M., Fahnrich S., Drexler H.G. (2019). Deregulated expression of NKL homeobox genes in T-cell lymphomas. Oncotarget.

[bib78] Oberlin E., Tavian M., Blazsek I., Peault B. (2002). Blood-forming potential of vascular endothelium in the human embryo. Development.

[bib79] Omatsu Y., Sugiyama T., Kohara H., Kondoh G., Fujii N., Kohno K., Nagasawa T. (2010). The essential functions of adipo-osteogenic progenitors as the hematopoietic stem and progenitor cell niche. Immunity.

[bib80] Oostendorp R.A.J., Harvey K.N., Kusadasi N., de Bruijn M.F.T.R., Saris C., Ploemacher R.E., Medvinsky A.L., Dzierzak E.A. (2002). Stromal cell lines from mouse aorta-gonads-mesonephros subregions are potent supporters of hematopoietic stem cell activity. Blood.

[bib81] Oostendorp R.A.J., Medvinsky A.J., Kusadasi N., Nakayama N., Harvey K., Orelio C., Ottersbach K., Covey T., Ploemacher R.E., Saris C., Dzierzak E. (2002). Embryonal subregion-derived stromal cell lines from novel temperature-sensitive SV40 T antigen transgenic mice support hematopoiesis. J. Cell Sci..

[bib82] Ottersbach K., Dzierzak E. (2005). The murine placenta contains hematopoietic stem cells within the vascular labyrinth region. Dev. Cell.

[bib83] Ozerdem U., Grako K.A., Dahlin-Huppe K., Monosov E., Stallcup W.B. (2001). NG2 proteoglycan is expressed exclusively by mural cells during vascular morphogenesis. Dev. Dyn..

[bib84] Paic F., Igwe J.C., Nori R., Kronenberg M.S., Franceschetti T., Harrington P., Kuo L., Shin D.G., Rowe D.W., Harris S.E., Kalajzic I. (2009). Identification of differentially expressed genes between osteoblasts and osteocytes. Bone.

[bib85] Perkhofer L., Walter K., Costa I.G., Carrasco M.C.R., Eiseler T., Hafner S., Genze F., Zenke M., Bergmann W., Illing A. (2016). Tbx3 fosters pancreatic cancer growth by increased angiogenesis and activin/nodal-dependent induction of stemness. Stem Cell Res..

[bib86] Picelli S., Bjorklund A.K., Faridani O.R., Sagasser S., Winberg G., Sandberg R. (2013). Smart-seq2 for sensitive full-length transcriptome profiling in single cells. Nat. Methods.

[bib87] Pijuan-Sala B., Griffiths J.A., Guibentif C., Hiscock T.W., Jawaid W., Calero-Nieto F.J., Mulas C., Ibarra-Soria X., Tyser R.C.V., Ho D.L.L. (2019). A single-cell molecular map of mouse gastrulation and early organogenesis. Nature.

[bib88] Pinho S., Lacombe J., Hanoun M., Mizoguchi T., Bruns I., Kunisaki Y., Frenette P.S. (2013). PDGFRα and CD51 mark human Nestin+ sphere-forming mesenchymal stem cells capable of hematopoietic progenitor cell expansion. J. Exp. Med..

[bib89] Porcheri C., Golan O., Calero-Nieto F.J., Thambyrajah R., Ruiz-Herguido C., Wang X., Catto F., Guillen Y., Sinha R., Gonzalez J. (2020). Notch ligand Dll4 impairs cell recruitment to aortic clusters and limits blood stem cell generation. EMBO J..

[bib90] Richard C., Drevon C., Canto P.Y., Villain G., Bollerot K., Lempereur A., Teillet M.A., Vincent C., Rossello Castillo C., Torres M. (2013). Endothelio-mesenchymal interaction controls runx1 expression and modulates the notch pathway to initiate aortic hematopoiesis. Dev. Cell.

[bib91] Rolny C., Nilsson I., Magnusson P., Armulik A., Jakobsson L., Wentzel P., Lindblom P., Norlin J., Betsholtz C., Heuchel R. (2006). Platelet-derived growth factor receptor-beta promotes early endothelial cell differentiation. Blood.

[bib92] Roostalu U., Aldeiri B., Albertini A., Humphreys N., Simonsen-Jackson M., Wong J.K.F., Cossu G. (2018). Distinct cellular mechanisms underlie smooth muscle turnover in vascular development and Repair. Circ. Res..

[bib93] Rosu-Myles M., She Y.M., Fair J., Muradia G., Mehic J., Menendez P., Prasad S.S., Cyr T.D. (2012). Identification of a candidate proteomic signature to discriminate multipotent and non-multipotent stromal cells. PLoS One.

[bib94] Ruiz-Herguido C., Guiu J., D'Altri T., Ingles-Esteve J., Dzierzak E., Espinosa L., Bigas A. (2012). Hematopoietic stem cell development requires transient Wnt/β-catenin activity. J. Exp. Med..

[bib95] Sá da Bandeira D., Casamitjana J., Crisan M. (2016). Pericytes, integral components of adult hematopoietic stem cell niches. Pharmacol. Ther..

[bib96] Sacchetti B., Funari A., Michienzi S., Di Cesare S., Piersanti S., Saggio I., Tagliafico E., Ferrari S., Robey P.G., Riminucci M., Bianco P. (2007). Self-renewing osteoprogenitors in bone marrow sinusoids can organize a hematopoietic microenvironment. Cell.

[bib97] Solaimani Kartalaei P., Yamada-Inagawa T., Vink C.S., de Pater E., van der Linden R., Marks-Bluth J., van der Sloot A., van den Hout M., Yokomizo T., van Schaick-Solerno M.L. (2015). Whole-transcriptome analysis of endothelial to hematopoietic stem cell transition reveals a requirement for Gpr56 in HSC generation. J. Exp. Med..

[bib98] Soriano P. (1994). Abnormal kidney development and Hematological disorders in Pdgf beta-receptor mutant mice. Gene Dev..

[bib99] Takimoto A., Kokubu C., Watanabe H., Sakuma T., Yamamoto T., Kondoh G., Hiraki Y., Shukunami C. (2019). Differential transactivation of the upstream aggrecan enhancer regulated by PAX1/9 depends on SOX9-driven transactivation. Sci. Rep..

[bib100] Tavian M., Robin C., Coulombel L., Peault B. (2001). The human embryo, but not its yolk sac, generates lympho-myeloid stem cells. Immunity.

[bib101] Trapnell C., Roberts A., Goff L., Pertea G., Kim D., Kelley D.R., Pimentel H., Salzberg S.L., Rinn J.L., Pachter L. (2012). Differential gene and transcript expression analysis of RNA-seq experiments with TopHat and Cufflinks. Nat. Protoc..

[bib102] Ulvmar M.H., Martinez-Corral I., Stanczuk L., Makinen T. (2016). Pdgfrb-Cre targets lymphatic endothelial cells of both venous and non-venous origins. Genesis.

[bib103] Vink C.S., Calero-Nieto F.J., Wang X., Maglitto A., Mariani S.A., Jawaid W., Gottgens B., Dzierzak E. (2020). Iterative single-cell analyses define the transcriptome of the first functional hematopoietic stem cells. Cell Rep..

[bib104] Washkowitz A.J., Gavrilov S., Begum S., Papaioannou V.E. (2012). Diverse functional networks of Tbx3 in development and disease. Wiley Interdiscip. Rev. Syst. Biol. Med..

[bib105] Wilkinson R.N., Pouget C., Gering M., Russell A.J., Davies S.G., Kimelman D., Patient R. (2009). Hedgehog and Bmp polarize hematopoietic stem cell emergence in the zebrafish dorsal aorta. Dev. Cell.

[bib106] Wright E., Hargrave M.R., Christiansen J., Cooper L., Kun J., Evans T., Gangadharan U., Greenfield A., Koopman P. (1995). The Sry-related gene Sox9 is expressed during chondrogenesis in mouse embryos. Nat. Genet..

[bib107] Yokomizo T., Dzierzak E. (2010). Three-dimensional cartography of hematopoietic clusters in the vasculature of whole mouse embryos. Development.

[bib108] Yokomizo T., Yamada-Inagawa T., Yzaguirre A.D., Chen M.J., Speck N.A., Dzierzak E. (2012). Whole-mount three-dimensional imaging of internally localized immunostained cells within mouse embryos. Nat. Protoc..

[bib109] Yvernogeau L., Klaus A., Maas J., Morin-Poulard I., Weijts B., Schulte-Merker S., Berezikov E., Junker J.P., Robin C. (2020). Multispecies RNA tomography reveals regulators of hematopoietic stem cell birth in the embryonic aorta. Blood.

[bib110] Yzaguirre A.D., Padmanabhan A., de Groh E.D., Engleka K.A., Li J., Speck N.A., Epstein J.A. (2015). Loss of neurofibromin Ras-GAP activity enhances the formation of cardiac blood islands in murine embryos. Elife.

[bib111] Zhu Q., Gao P., Tober J., Bennett L., Chen C., Uzun Y., Li Y., Howell E.D., Mumau M., Yu W. (2020). Developmental trajectory of prehematopoietic stem cell formation from endothelium. Blood.

[bib112] Zovein A.C., Hofmann J.J., Lynch M., French W.J., Turlo K.A., Yang Y., Becker M.S., Zanetta L., Dejana E., Gasson J.C. (2008). Fate tracing reveals the endothelial origin of hematopoietic stem cells. Cell Stem Cell.

