# Supplementary material for: PDGFRβ+ cells play a dual role as hematopoietic precursors and niche cells during mouse ontogeny
Source: Cell Rep. 2022 Jul 19;40(3):111114. doi: 10.1016/j.celrep.2022.111114 (PMC9638014; doi:10.1016/j.celrep.2022.111114)
Supplement: Document S1. Figures S1–S7 and Tables S1–S7 [file mmc1.pdf]

Cell Reports, Volume *40*

Supplemental information

PDGFRβ^{+} cells play a dual role as hematopoietic precursors and niche cells during mouse ontogeny

Diana Sá da Bandeira, Alastair Morris Kilpatrick, Madalena Marques, Mario Gomez-Salazar, Telma Ventura, Zaniah Nashira Gonzalez, Dorota Stefancova, Fiona Rossi, Matthieu Vermeren, Chris Sebastiaan Vink, Mariana Beltran, Neil Cowan Henderson, Bongnam Jung, Reinier van der Linden, Harmen Jan George van de Werken, Wilfred F.J. van Ijcken, Christer Betsholtz, Stuart John Forbes, Henar Cuervo, and Mihaela Crisan

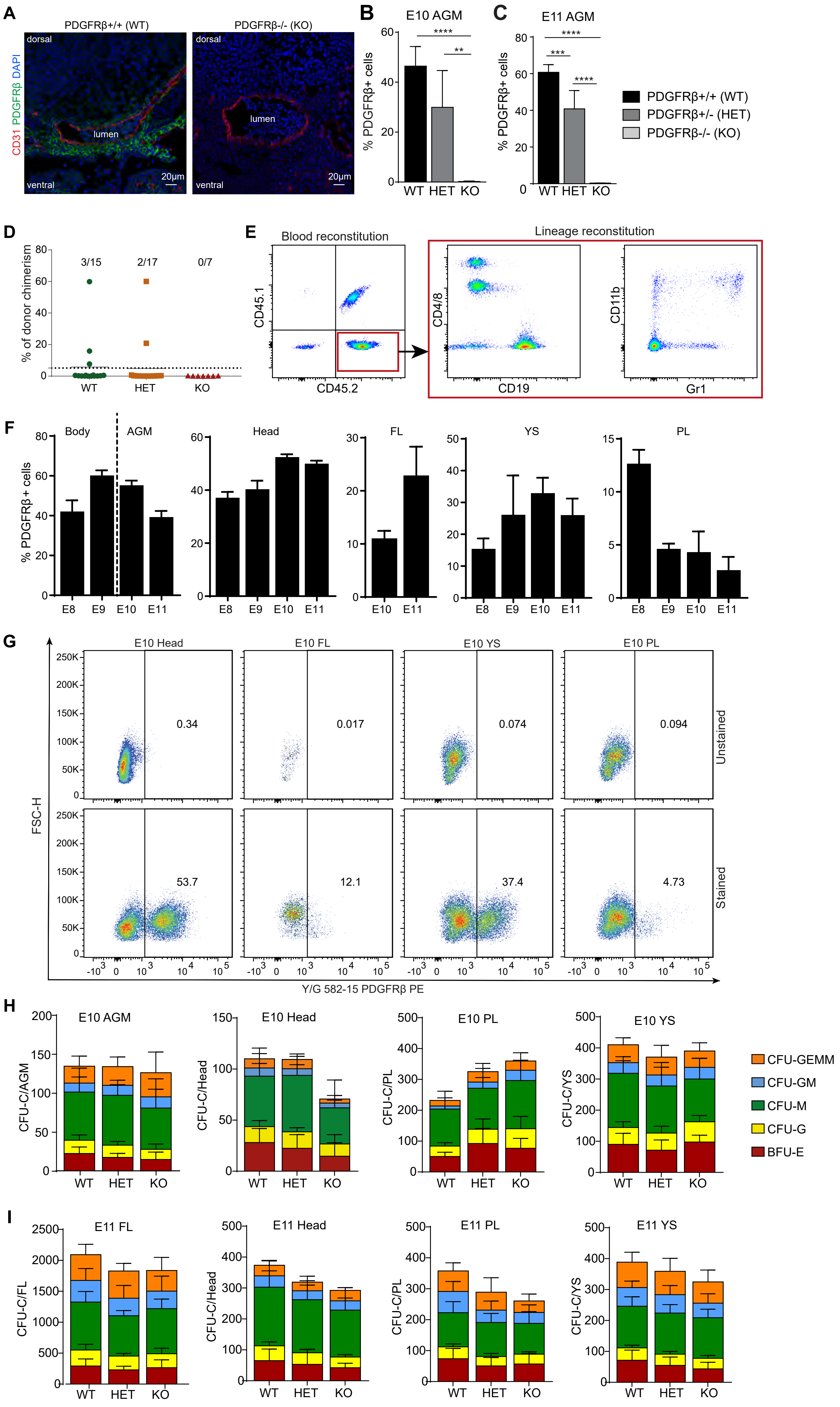

A
PDGFRβ+/+ (WT)
PDGFRβ-/- (KO)
CD31 PDGFRβ DAPI
dorsal
lumen
ventral
20μm
B
E10 AGM
% PDGFRβ+ cells
WT HET KO
C
E11 AGM
PDGFRβ+/+ (WT)
PDGFRβ+/- (HET)
PDGFRβ-/- (KO)
D
% of donor chimerism
3/15
2/17
0/7
WT
HET
KO
E
Blood reconstitution
Lineage reconstitution
CD45.1
CD45.2
CD4/8
CD19
CD11b
Gr1
F
Body
AGM
Head
FL
YS
PL
% PDGFRβ+ cells
E8 E9 E10 E11
G
E10 Head
E10 FL
E10 YS
E10 PL
0.34
0.017
0.074
0.094
Unstained
53.7
12.1
37.4
4.73
Stained
FSC-H
Y/G 582-15 PDGFRβ PE
H
E10 AGM
E10 Head
E10 PL
E10 YS
CFU-C/AGM
CFU-C/Head
CFU-C/PL
CFU-C/YS
CFU-GEMM
CFU-GM
CFU-M
CFU-G
BFU-E
I
E11 FL
E11 Head
E11 PL
E11 YS
CFU-C/FL

Figure S1. Hematopoietic stem/progenitor activity is not affected in all PDGFRβ-KO embryonic organs, related to Figure 2. (A) Immunohistochemistry showing PDGFRβ and CD31 expression on E11 AGM in PDGFRβ WT/KO AGM. Flow cytometric analysis of PDGFRβ+ cells in PDGFRβ mutants at (B) E10, 33-36sp, WT/HET/KO: 6/17/8 embryos and (C) E11, 43-46sp, WT/HET/KO: 5/7/7 embryos. Error bars: SD. **p<0.01, ***p<0.001, ****p<0.0001. One-way ANOVA or Kruskal-Wallis tests were used depending on the data distribution. (D) Long-term reconstitution potential of E11 PDGFRβ mutant AGMs 4 months after primary transplantations. Each data point represents one mouse injected with 1 embryo equivalent. Mice positively reconstitute when >5% donor cells (dashed line) are found in the host peripheral blood. Numbers of reconstituted mice/number of transplanted mice are given in each group. (E) Flow cytometry analysis showing an example of positive, multi-lineage reconstitution of a mouse injected with E11 WT AGM donor cells, 4 months post-transplantation. (F) Percentage of PDGFRβ+ cells in WT (C57Bl6) hematopoietic organs from E8 to E11, determined by flow cytometry. E10 AGM (n=6); E11 AGM (n=7); n=3 for all the other organs and developmental stages. (G) Representative example of the flow cytometry gating used to analyse PDGFRβ in E10 organs. (H-I): CFU-C numbers per PDGFRβ+/+ (WT), PDGFRβ+/- (HET) and PDGFRβ-/- (KO) (H) E10 (Table S2) and (I) E11 (Table S3).

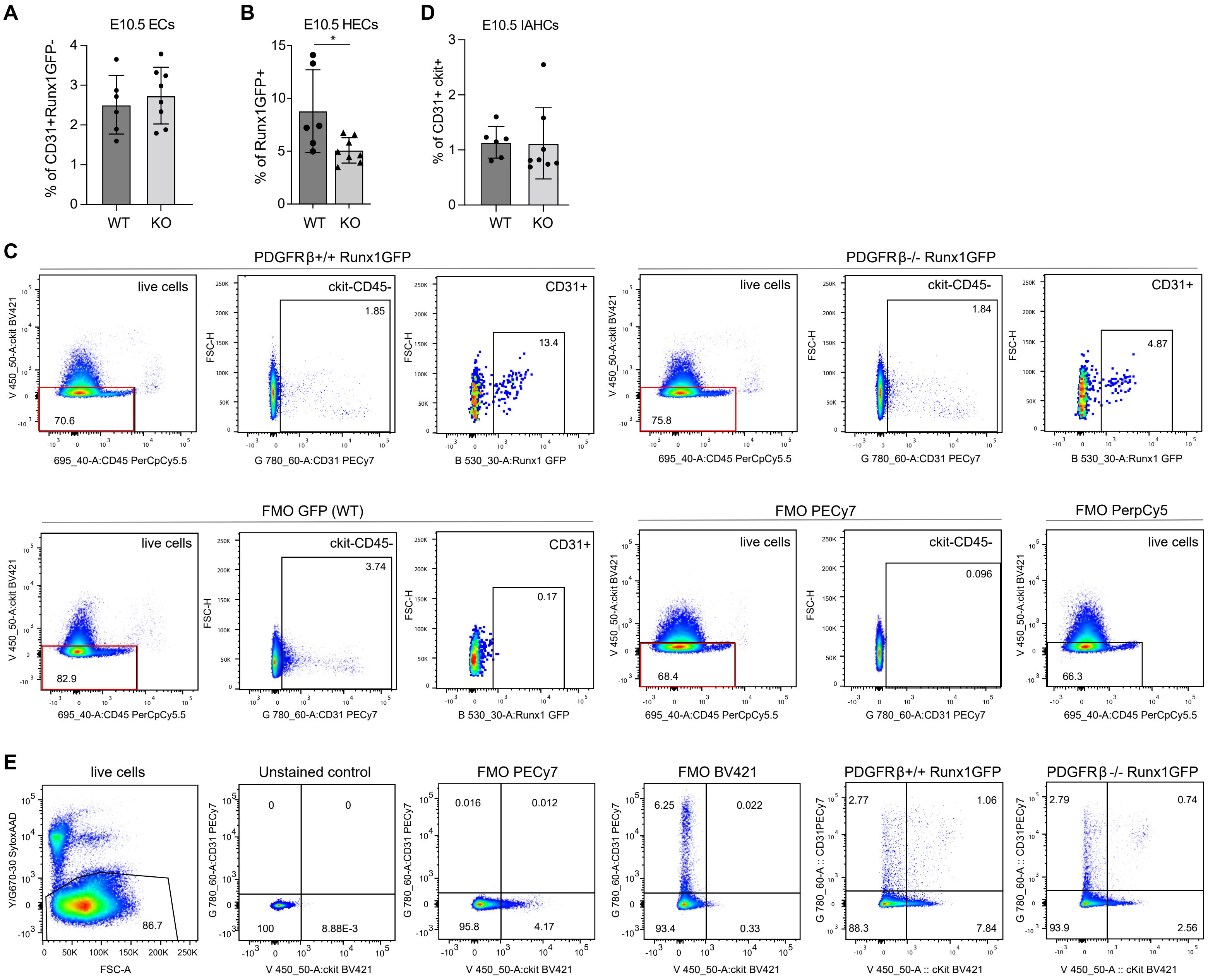

Figure S2. Comparison between PDGFRβ+/+ (WT) and PDGFRβ-/- (KO) AGMs by flow cytometry, related to Figure 2. Flow cytometry analysis gating example and controls and comparison between WT (n=6) and KO (n=8) ECs (A), HEC/EHT cells (B-C) and IAHCs (D-E) frequencies in E10.5 AGMs (33-36sp) obtained from PDGFRβ;Runx1gfp/gfp mouse embryos. To aid visibility, rare populations are shown with large dots. Error bars: mean ± SD. *p=0.0252 (HEC/EHT) by unpaired t-test.

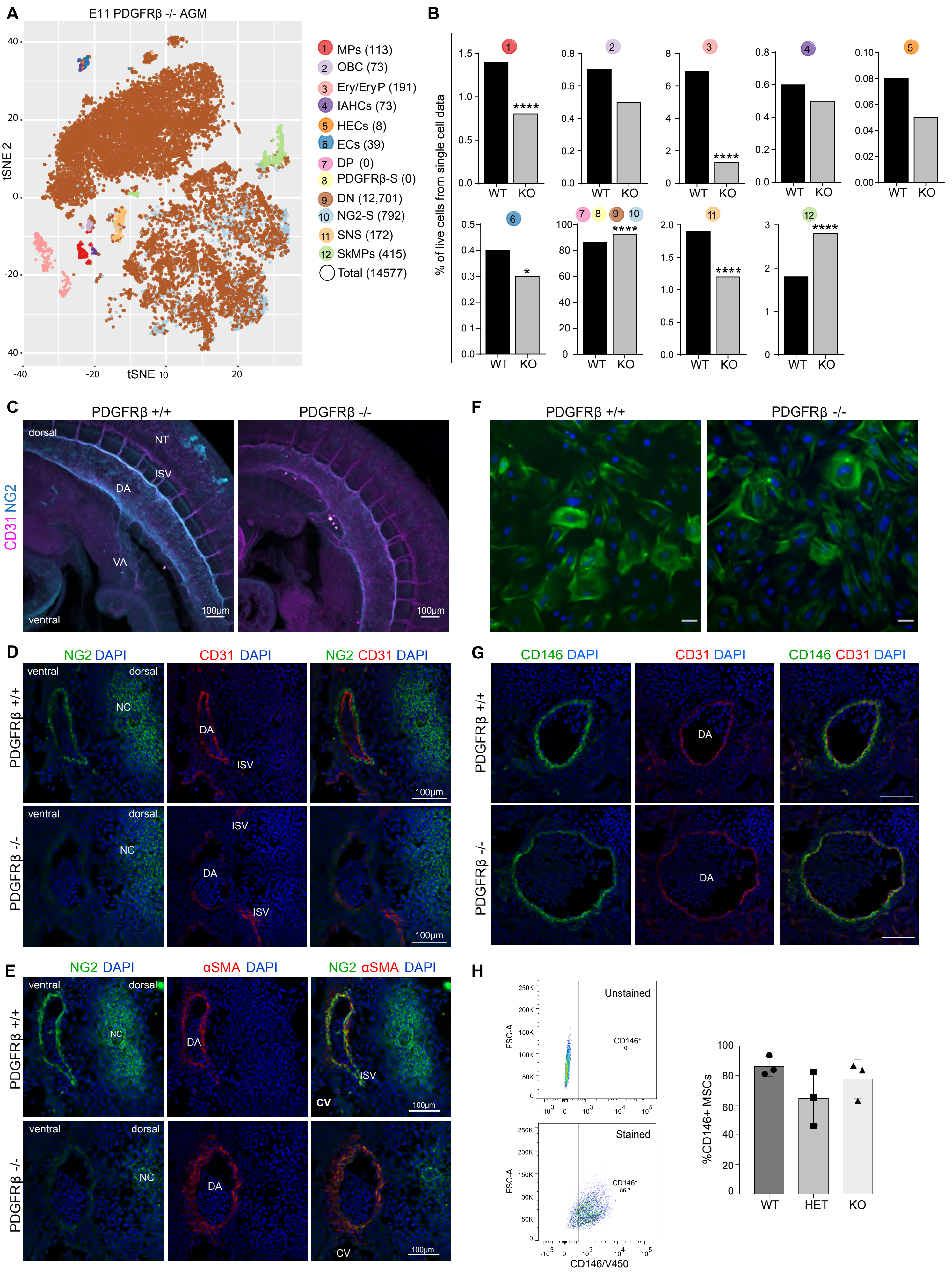

A
E11 PDGFRβ -/- AGM
tSNE 2
tSNE 10
MPs (113)
OBC (73)
Ery/EryP (191)
IAHCs (73)
HECs (8)
ECs (39)
DP (0)
PDGFRβ-S (0)
DN (12,701)
NG2-S (792)
SNS (172)
SkMPs (415)
Total (14577)
B
% of live cells from single cell data
WT
KO
C
PDGFRβ +/+
PDGFRβ -/-
CD31 NG2
dorsal
ventral
NT
ISV
DA
VA
100µm
D
NG2 DAPI
CD31 DAPI
NG2 CD31 DAPI
NC
E
αSMA DAPI
NG2 αSMA DAPI
CV
F
G
CD146 DAPI
CD146 CD31 DAPI
H
Unstained
Stained
FSC-A
CD146/V450
CD146+
86,7
%CD146+ MSCs
WT
HET
KO

Figure S3. Imaging and scRNA-seq analysis of PDGFRβ+/+ (WT) and PDGFRβ-/- (KO) AGM, related to Figures 2 and 3. (A) t-SNE plots showing 10 cell populations found in the E11 KO AGM (42sp). Each dot represents one cell and colors represent cell populations as indicated. In the absence of PDGFRβ, DP (PDGFRβ+NG2+) and PDGFRβ-S (PDGFRβ+NG2-) clusters 7 and 8 are absent. The number of cells in each population is shown between brackets. MP, macrophages; OBC, other blood cells; Ery/EryP, erythroid/progenitors; IAHC/HSPC, intra-aortic hematopoietic clusters/hematopoietic stem/progenitor cells; HEC/EHT, hemogenic endothelial cells/endothelial-to-hematopoietic transition, EC, endothelial cells; SNS, sympathetic nervous system; SkMP, skeletal muscle progenitors. (B) Percentage of single live cells found in each E11 AGM population (cell number/total cells) defined by scRNA-seq in PDGFRβ WT/KO AGMs. ****$p<0.0001$, *$p=0.015$, Fisher's Exact Test. (C) Confocal whole-mount immunofluorescence of WT and KO E10.5 dorsal aorta stained with CD31 and NG2. (D-E) Immunohistochemistry on frozen transversal sections of E11 WT and KO dorsal aorta with NG2 (green) and (D) CD31 (red) or (E) αSMA (red). (F) Immunocytochemistry using αSMA antibody (green) and DAPI (blue) on WT and KO MSCs. (G) Immunohistochemistry on frozen transversal sections of E11 WT and KO dorsal aorta with CD146 (green), CD31 (red), and DAPI (blue). (H). Flow cytometry analysis of WT, HET and KO MSCs using CD146 antibody (WT/HET/KO = 3/3/3). An example of gating of unstained and stained cells is shown.

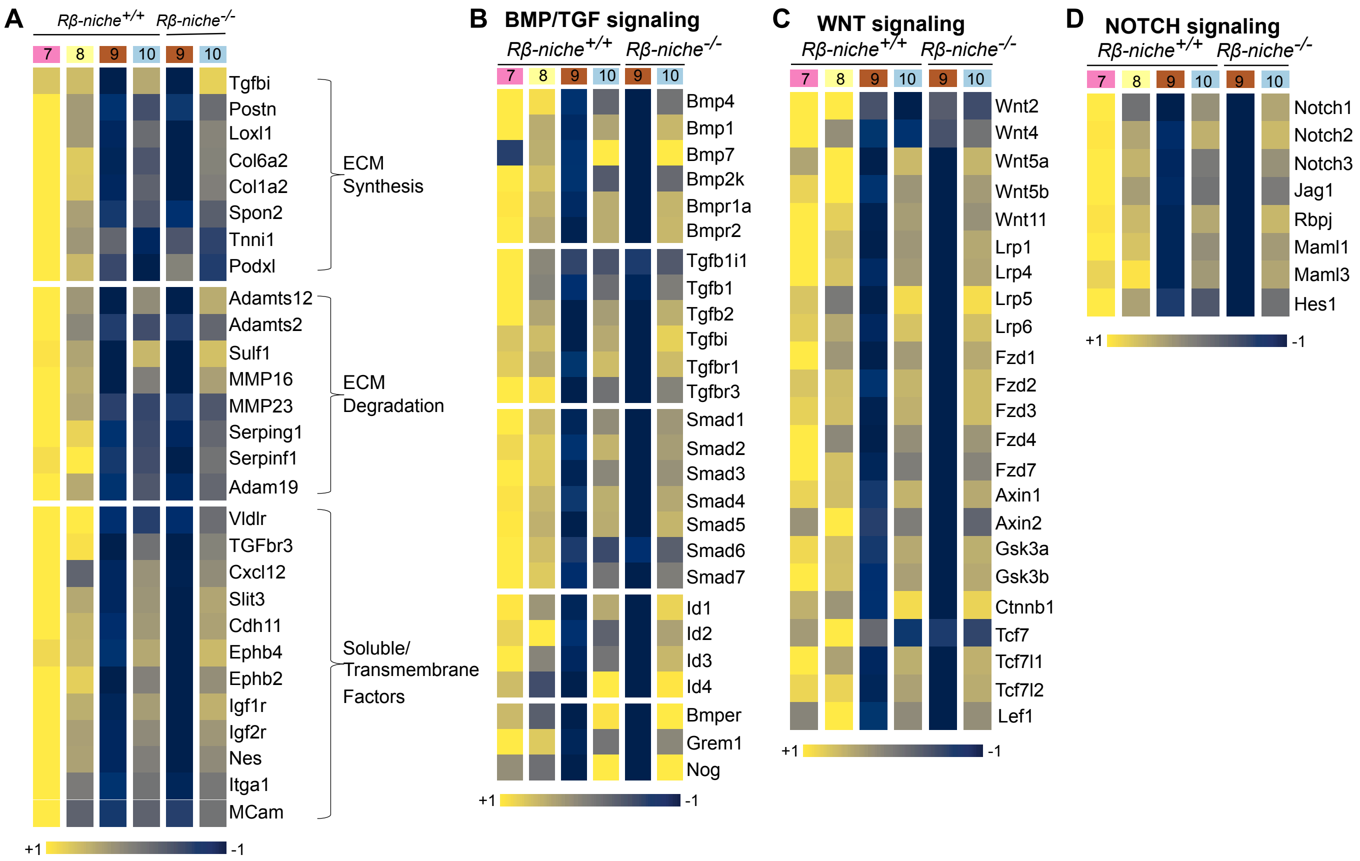

Figure S4. Transcriptomic differences by scRNA-seq between WT and KO Rβ-niche, related to Figure 2. (A) Heatmap showing selected upregulated genes in hematopoietic-supportive AGM-derived stromal clones (Charbord et al., 2014) that are significantly downregulated in the KO Rβ-niche cluster compared to the WT Rβ-niche (Table S5a). Heatmaps showing genes associated with BMP/TGF (B), WNT (C) and NOTCH (D) signaling pathways significantly downregulated in the Rβ-niche AGM KO cluster compared to PDGFRβ WT littermate control (Table S5c-e). Colors in boxes show the average expression of genes in each cluster.

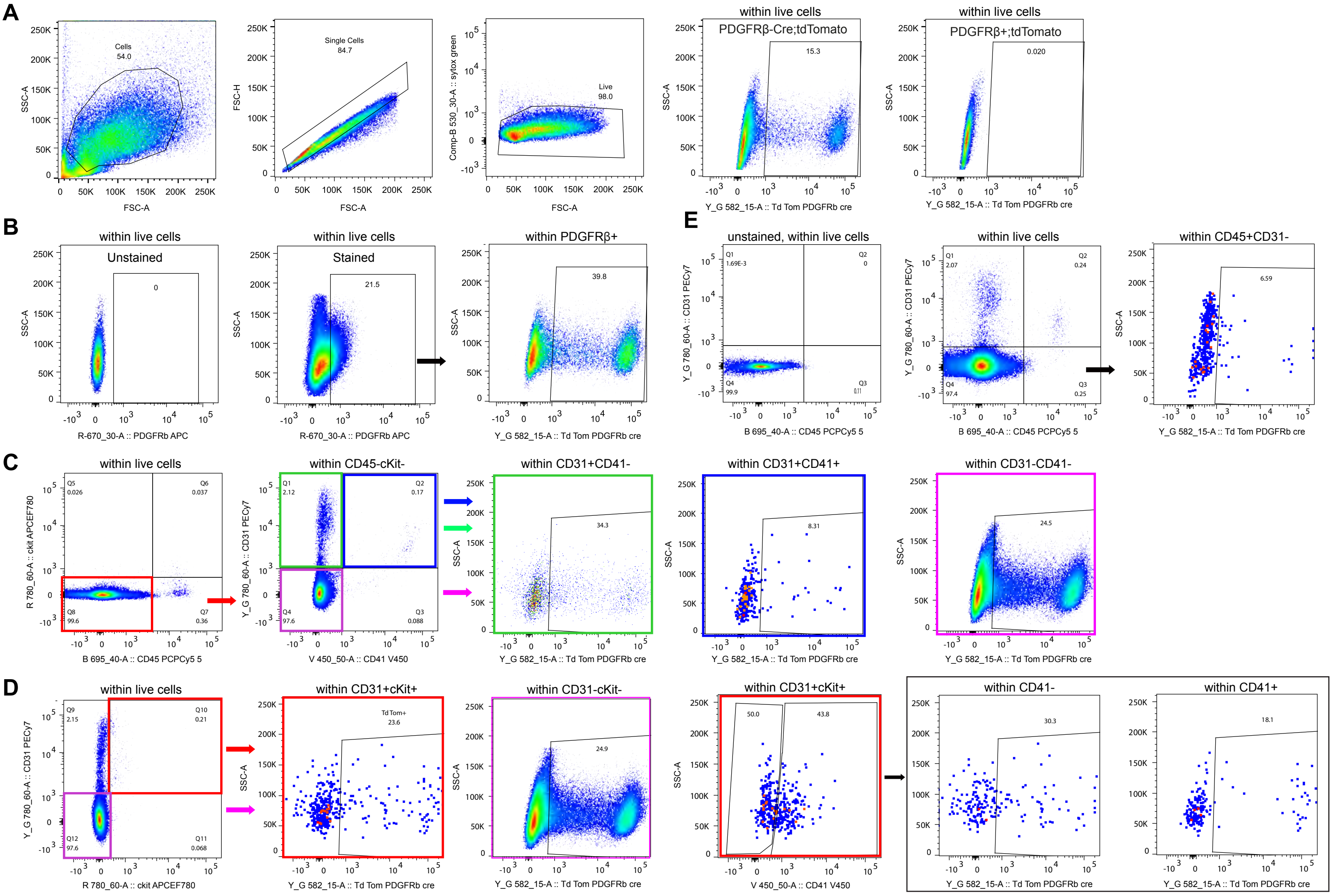

F

Population	Gene	Cell number expressing the gene	Max normalised log counts	Mean normalised log expression of the gene over this population	Cells with positive doublet score
DP	*Pdgfrb*	466/466	3.26	1.27	
7	*Pecam1*	13*/466	1.38	0.02	*13 cells
	Ptprc	4*/466	0.47	0.002	*4 cells
PDGFRβ-S	*Pdgfrb*	2371/2371	5.28	1.35	
8	*Pecam1*	46*/2371	2.88	0.02	*45 cells
	Ptprc	11*/2371	1.37	0.003	*11 cells
EC	*Pdgfrb*	0	0	0	
6	*Pecam1*	43/46	4.57	2.79	
	Ptprc	0	0	0	
HEC/EHT	*Pdgfrb*	2*/8	0.25/0.64*	0.11	*1 cell (0.64)
5	*Pecam1*	8/8	2.86	2.32	
	Ptprc	1*/8	0.25	0.03	*1 cell
IAHC	*Pdgfrb*	5*/63	1.25*	0.05	*5 cells
4	*Pecam1*	51/63	4.08	1.94	
	Ptprc	16/63	3.32	0.49	

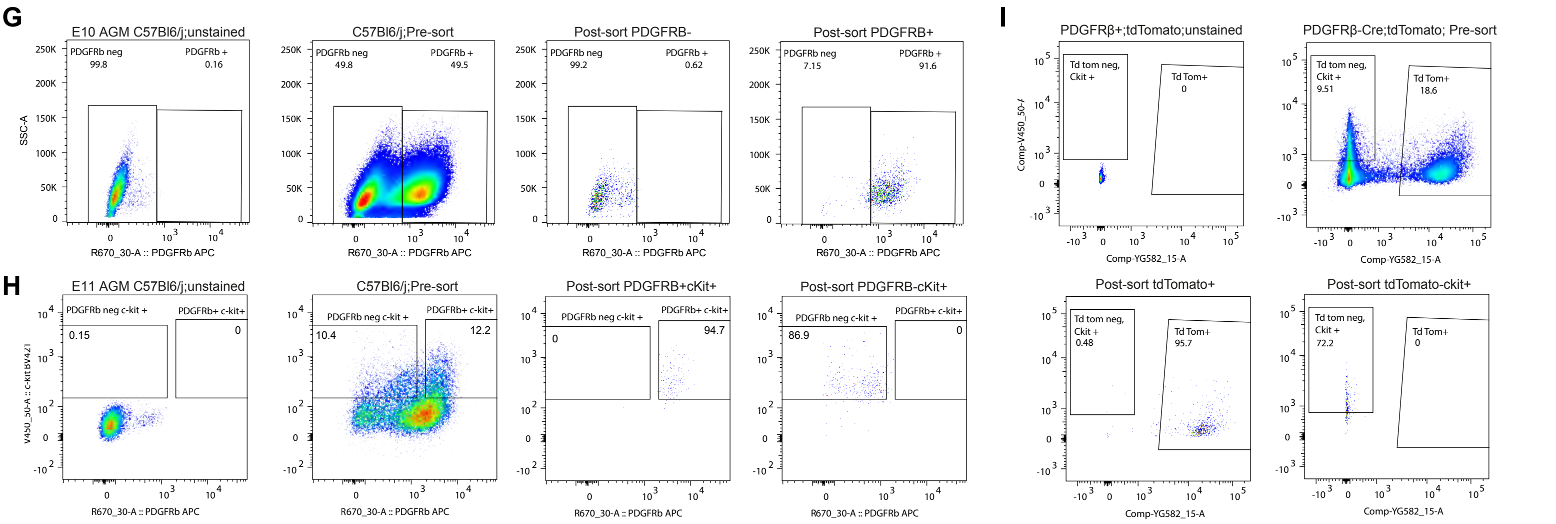

Figure S5. Flow cytometry analysis and cell sorting, related to Figures 1, 5 and 6. Representative examples of the gating strategies showing (A) tdTomato expression in PDGFRβ-Cre;tdTomato E10 AGM live cells and +;tdTomato WT control littermates. tdTomato expression in (B) PDGFRβ+ cells, (C) CD45-ckit-CD31+CD41- ECs, CD45-ckit-CD31+CD41+ HEC/EHT cells, and CD45-ckit-CD31-CD41- stromal cells, (D) IAHCs/HPSCs (CD31+ckit+) and other cells (CD31-ckit-), and (E) MPs (CD45+CD31-) in the E10 AGM. All gates were based on unstained and full/fluorescence minus one (FMO) controls in tdTomato and WT littermates. (F) Expression of *Pdgfrb*, *Pecam1* and *Ptprc* in various populations by scRNA-seq in WT AGM and doublet score analysis. (G-I) Representative example of PDGFRβ+/- cell sorting and post-sorting purity checks from E10 (G) and E11 C57BL/6J WT AGM (H). E11 AGM cells were further enriched in ckit+ (H). (I) Representative example of tdTomato+/- cell sorting and post-sorting purity checks from E11 PDGFRβ-Cre;tdTomato AGM. E11 tdTomato- cell fraction was further enriched in ckit+. All gates were based on unstained and FMO controls. To aid visibility, rare populations are shown with large dots.

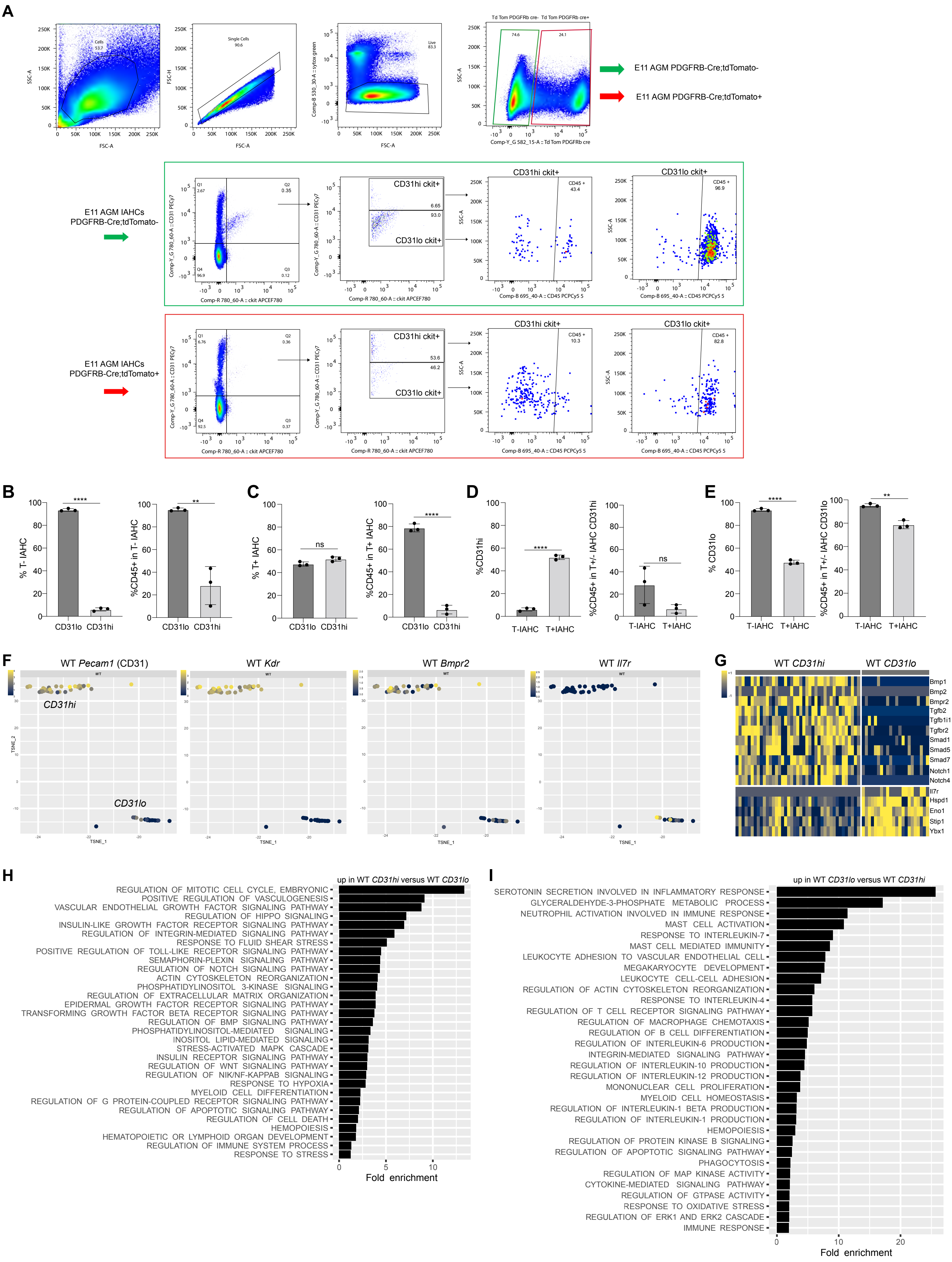

Sa da Bandeira et al, R2 revised Figure S6

Figure S6. E11 AGM PDGFRβCre:tdTomato+ IAHCs are transcriptionally and phenotypically different, related to Figure 5. Representative examples of the gating strategies showing T+ and T- IAHC/HSPCs (CD31+ckit+), CD31hi and CD31lo then CD45+ cells (A) and statistical comparison between various populations (B-E); n=3, **$p<0.01$, ****$p<0.0001$, unpaired t test, ns = not significant. Large dots are used to show the most rare population to aid visibility. (F-G) Identification of Pecam1hi (CD31hi) and Pecam1lo (CD31lo) sub-clusters by scRNA-seq and example of selected gene expression (F) and heatmap showing gene expressions associated with BMP/TGFβ, NOTCH, WNT and IL7 signaling pathways in each sub-cluster at single cell level (G) in the WT. (H-I) Selected Gene Ontology (GO) biological processes significantly overrepresented in genes significantly upregulated in the WT CD31hi IAHC (H) and in the WT CD31lo IAHC (I) sub-clusters.

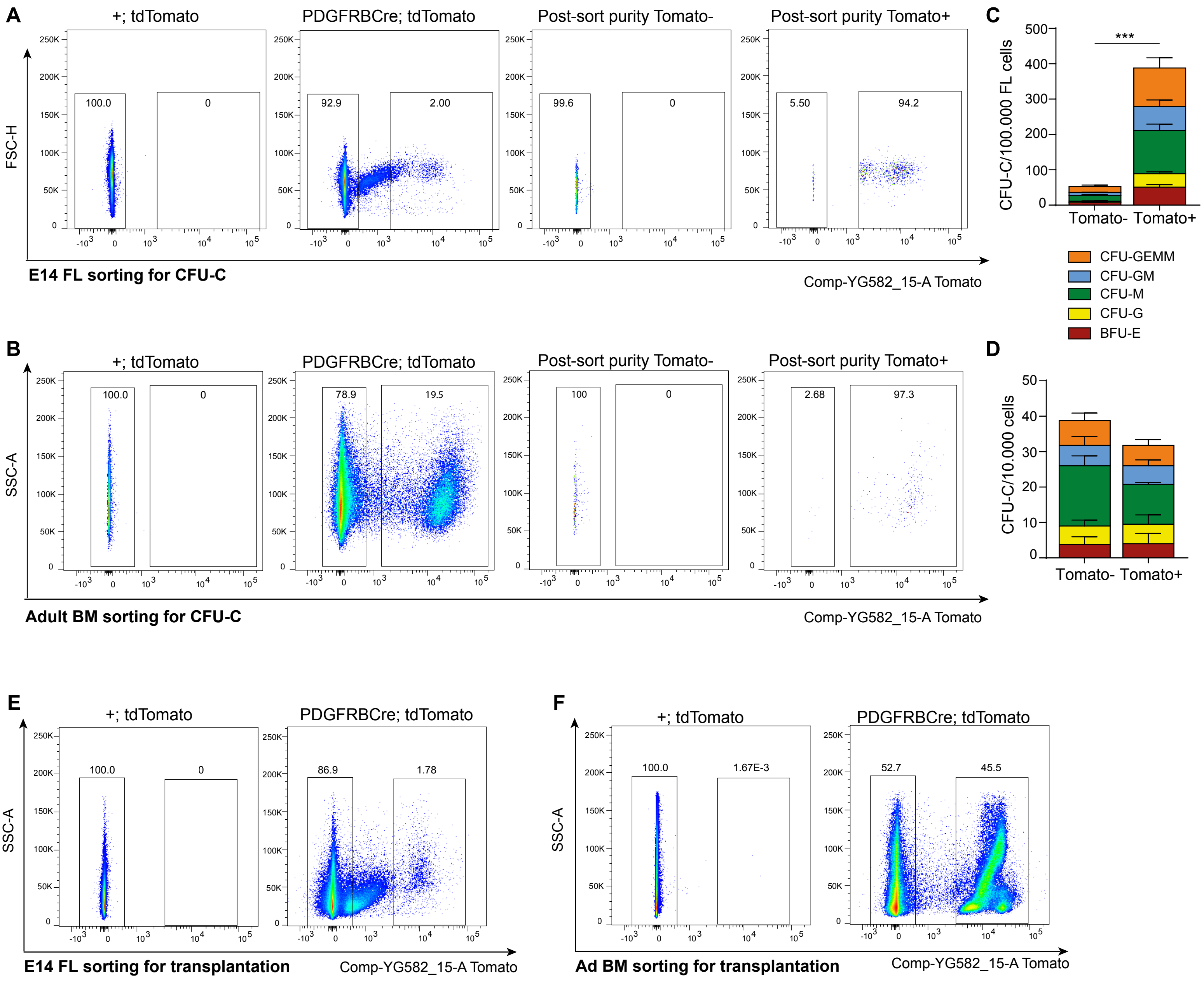

Figure S7. Flow cytometry analysis of E14FL and adult BM in PDGFRβCre:tdTomato mice, related to Figure 7. Representative example of Tomato+ and Tomato- cell sorting and post-sort purity check from E14FL (A) and adult BM (B) that were seeded in CFU-C in vitro assays. CFU-C frequencies in Tomato+ and Tomato- cells sorted from E14FL (C, n=3) and adult BM (D, n=4). Error bars: SD; ***$p<0.001$. Unpaired student t-test, unpaired student t-test with Welch correction or Mann-Whitney U test were used depending on data distribution and equality of variance. Example of Tomato+ and Tomato- cell sorting from E14 FL (E) and adult BM (F) that were transplanted into irradiated mice.

Table S1. Summary of CFU-C data obtained from PDGFRβ WT, HET and KO E11 AGMs and statistical analyses performed. Related to Figure 2

				Number of embryos			Average			Fold decrease							Multiple comparisons		
Tissue	Day	Somite pairs	CFU-type	WT	HET	KO	WT	HET	KO	WT/HET	WT/KO	HET/KO	Statistical test	p-value		Post-hoc	WT/HET	WT/KO	Het/KO
AGM	11	40-45	BFU-E	14	17	8	27.50	17.59	14.00	1.56	1.96	1.26	1-way ANOVA	0.0042	**	Tukey's	*	**	ns
			CFU-G				28.43	19.94	13.25	1.43	2.15	1.50	Kruskal-Wallis	0.0002	***	Dunn's	*	***	ns
			CFU-M				102.64	94.24	67.88	1.09	1.51	1.39	1-way ANOVA	0.0171	*	Tukey's	ns	*	ns
			CFU-GM				24.93	16.71	11.13	1.49	2.24	1.50	Kruskal-Wallis	0.0254	*	Dunn's	ns	*	ns
			CFU-GEMM				30.14	21.53	14.25	1.40	2.12	1.51	1-way ANOVA	0.0129	*	Tukey's	ns	*	ns
			Total				213.64	170.00	120.50	1.26	1.77	1.41	1-way ANOVA	0.0002	***	Tukey's	*	***	*

Table S2. Summary of CFU-C data obtained from PDGFRβ WT, HET and KO E10 hematopoietic organs and statistical analyses performed. Related to Fig 1, S1

				Number of embryos			Average			Fold decrease							Multiple comparisons		
Tissue	Day	Somite pairs	CFU-type	WT	HET	KO	WT	HET	KO	WT/HET	WT/KO	HET/KO	Statistical test	p-value		Post-hoc	WT vs HET	WT vs KO	Het vs KO
AGM	10	31-34	BFU-E	7	27	3	22.71	17.74	15.00	1.28	1.51	1.18	1-way ANOVA	0.1145	ns	Tukey's	ns	ns	ns
			CFU-G				17.00	15.59	13.00	1.09	1.31	1.20	1-way ANOVA	0.5749	ns	Tukey's	ns	ns	ns
			CFU-M				61.86	64.04	53.00	0.97	1.17	1.21	1-way ANOVA	0.6564	ns	Tukey's	ns	ns	ns
			CFU-GM				11.43	12.89	14.33	0.89	0.80	0.90	1-way ANOVA	0.815	ns	Tukey's	ns	ns	ns
			CFU-GEMM				21.71	24.00	31.00	0.90	0.70	0.77	Kruskal-Wallis	0.7387	ns	Dunn's	ns	ns	ns
			Total				134.71	134.26	126.33	1.00	1.07	1.06	1-way ANOVA	0.9569	ns	Tukey's	ns	ns	ns
Head	10	31-34	BFU-E	7	27	3	28.00	22.44	14.67	1.25	1.91	1.53	Kruskal-Wallis	0.304	ns	Dunn's	ns	ns	ns
			CFU-G				15.67	16.15	12.00	0.97	1.31	1.35	1-way ANOVA	0.373	ns	Tukey's	ns	ns	ns
			CFU-M				49.33	55.19	35.33	0.89	1.40	1.56	1-way ANOVA	0.2826	ns	Tukey's	ns	ns	ns
			CFU-GM				8.00	6.52	4.67	1.23	1.71	1.40	Kruskal-Wallis	0.4811	ns	Dunn's	ns	ns	ns
			CFU-GEMM				9.00	9.11	4.00	0.99	2.25	2.28	Kruskal-Wallis	0.2554	ns	Dunn's	ns	ns	ns
			Total				110.00	109.41	70.67	1.01	1.56	1.55	1-way ANOVA	0.2519	ns	Tukey's	ns	ns	ns
PL	10	31-34	BFU-E	5	26	3	50.00	92.31	76.67	0.54	0.65	1.20	1-way ANOVA	0.1646	ns	Tukey's	ns	ns	ns
			CFU-G				34.00	46.15	63.33	0.74	0.54	0.73	Kruskal-Wallis	0.5557	ns	Dunn's	ns	ns	ns
			CFU-M				120.00	133.08	156.67	0.90	0.77	0.85	Kruskal-Wallis	0.4175	ns	Dunn's	ns	ns	ns
			CFU-GM				10.00	20.00	33.33	0.50	0.30	0.60	Kruskal-Wallis	0.0995	ns	Dunn's	ns	ns	ns
			CFU-GEMM				18.00	33.46	30.00	0.54	0.60	1.12	Kruskal-Wallis	0.5791	ns	Dunn's	ns	ns	ns
			Total				232.00	325.00	360.00	0.71	0.64	0.90	Kruskal-Wallis	0.4289	ns	Dunn's	ns	ns	ns
YS	10	31-34	BFU-E	7	27	4	90.00	71.48	97.50	1.26	0.92	0.73	1-way ANOVA	0.1911	ns	Tukey's	ns	ns	ns
			CFU-G				54.29	55.19	65.00	0.98	0.84	0.85	1-way ANOVA	0.6748	ns	Tukey's	ns	ns	ns
			CFU-M				174.29	151.11	137.50	1.15	1.27	1.10	1-way ANOVA	0.6542	ns	Tukey's	ns	ns	ns
			CFU-GM				34.29	35.19	37.50	0.97	0.91	0.94	Kruskal-Wallis	0.97	ns	Dunn's	ns	ns	ns
			CFU-GEMM				57.14	57.78	52.50	0.99	1.09	1.10	Kruskal-Wallis	0.9716	ns	Dunn's	ns	ns	ns
			Total				410.00	370.74	390.00	1.11	1.05	0.95	1-way ANOVA	0.8235	ns	Tukey’s	ns	ns	ns

Table S3. Summary of CFU-C data for PDGFRβ WT, HET, KO E11 hematopoietic organs (excluding AGM) and statistical analyses performed. Related to Fig1, S1

				Embryos			Average			Fold decrease							Multiple comparisons		
Tissue	Day	Somite pairs	CFU-type	WT	HET	KO	WT	HET	KO	WT/HET	WT/KO	HET/KO	Test	p-value		Posthoc	WT/HET	WT/KO	Het/KO
Head	11	40-45	BFU-E	14	18	7	65.00	53.06	42.29	1.23	1.54	1.25	Kruskal-Wallis	0.5005	ns	Dunn's	ns	ns	ns
			CFU-G				48.07	37.50	34.71	1.28	1.38	1.08	1-way ANOVA	0.013	*	Tukey's	*	*	ns
			CFU-M				189.71	171.61	151.71	1.11	1.25	1.13	1-way ANOVA	0.5263	ns	Tukey's	ns	ns	ns
			CFU-GM				36.50	28.83	30.00	1.27	1.22	0.96	1-way ANOVA	0.4112	ns	Tukey's	ns	ns	ns
			CFU-GEMM				34.14	28.11	33.43	1.21	1.02	0.84	1-way ANOVA	0.534	ns	Tukey's	ns	ns	ns
			Total				373.43	319.11	292.14	1.17	1.28	1.09	Kruskal-Wallis	0.213	ns	Dunn's	ns	ns	ns
FL	11	40-45	BFU-E	14	17	7	292.86	229.41	265.71	1.28	1.10	0.86	Kruskal-Wallis	0.3395	ns	Dunn's	ns	ns	ns
			CFU-G				258.57	224.71	225.71	1.15	1.15	1.00	Kruskal-Wallis	0.8322	ns	Dunn's	ns	ns	ns
			CFU-M				774.29	650.59	728.57	1.19	1.06	0.89	Kruskal-Wallis	0.0361	*	Dunn's	ns	ns	ns
			CFU-GM				350.00	284.71	282.86	1.23	1.24	1.01	Kruskal-Wallis	0.215	ns	Dunn's	ns	ns	ns
			CFU-GEMM				417.14	438.82	334.29	0.95	1.25	1.31	1-way ANOVA	0.3418	ns	Tukey's	ns	ns	ns
			Total				2092.86	1828.24	1837.14	1.14	1.14	1.00	Kruskal-Wallis	0.1863	ns	Dunn's	ns	ns	ns
PL	11	40-45	BFU-E	13	18	7	73.92	50.78	56.86	1.46	1.30	0.89	1-way ANOVA	0.1228	ns	Tukey's	ns	ns	ns
			CFU-G				38.23	30.33	31.57	1.26	1.21	0.96	1-way ANOVA	0.0825	ns	Tukey's	ns	ns	ns
			CFU-M				110.38	109.89	99.71	1.00	1.11	1.10	1-way ANOVA	0.7581	ns	Tukey's	ns	ns	ns
			CFU-GM				69.00	39.89	35.43	1.73	1.95	1.13	Kruskal-Wallis	0.0291	*	Dunn's	*	ns	ns
			CFU-GEMM				66.23	58.06	37.14	1.14	1.78	1.56	1-way ANOVA	0.2594	ns	Tukey's	ns	ns	ns

			Total				357.77	288.94	281.14	1.24	1.27	1.03	Kruskal-Wallis	0.1231	ns	Dunn's	ns	ns	ns
YS	11	40-45	BFU-E	7	27	4	71.36	54.83	43.63	1.30	1.64	1.26	Kruskal-Wallis	0.0428	*	Dunn's	ns	*	ns
			CFU-G				40.21	36.39	33.88	1.11	1.19	1.07	Kruskal-Wallis	0.2351	ns	Dunn's	ns	ns	ns
			CFU-M				134.29	132.67	131.38	1.01	1.02	1.01	1-way ANOVA	0.9724	ns	Tukey's	ns	ns	ns
			CFU-GM				60.79	59.50	47.00	1.02	1.29	1.27	1-way ANOVA	0.4766	ns	Tukey's	ns	ns	ns
			CFU-GEMM				81.93	75.56	68.88	1.08	1.19	1.10	1-way ANOVA	0.7383	ns	Tukey's	ns	ns	ns
			Total				388.57	358.94	324.75	1.08	1.20	1.11	1-way ANOVA	0.0934	ns	Tukey's	ns	ns	ns

Table S4. Details of genes shown in heatmaps. Related to Figures 2, 3, and 5.

Percentages and absolute cell numbers of DP and NG2-S WT cells expressing the gene (Figure 1D).

**Gene**	**% WT DP cells expressing the gene**	**% WT NG2-S cells expressing the gene**	**Whole number of WT DP cells expressing the gene** (total DP=466 cells)	**Whole number of WT NG2-S cells expressing the gene** (total NG2-S=285 cells)	**Ratio** DP/ DP+NG2-S
Rgs5	**51.72**	**13.33**	**241.02**	**37.99**	**0.86**
Mcam	42.06	12.98	196.00	36.99	0.84
Itga1	41.2	12.98	191.99	36.99	0.84
Jag1	50	18.25	233.00	52.01	0.82
Itga4	28.76	10.53	134.02	30.01	0.82
Notch1	43.56	18.25	202.99	52.01	0.80
Cd248	78.11	34.74	363.99	99.01	0.79
Notch3	66.52	33.33	309.98	94.99	0.77
Igf2r	88.2	63.16	411.01	180.01	0.70
Ncam1	89.7	68.42	418.00	195.00	0.68

Genes shown in Figure S4A.

**Symbol**	**AUC**	**Multiple comparisons (FDR)**	
Tgfbi	0.5521	3.67E-21	****
Postn	0.5522	2.78E-21	****
Loxl1	0.5399	7.10E-13	****
Col6a2	0.5261	3.83E-06	****
Col1a2	0.6209	1.21E-108	****
Spon2	0.5193	8.10E-04	***
Tnni1	0.5418	4.89E-14	****
Podxl	0.5232	4.77E-05	****
Adamts12	0.5242	2.09E-05	****
Adamts2	0.5221	1.13E-04	****
Sulf1	0.5342	8.66E-10	****
Mmp16	0.5420	3.49E-14	****
Mmp23	0.5470	1.86E-17	****
Serping1	0.5426	1.50E-14	****
Serpinf1	0.6094	3.56E-89	****
Adam19	0.5603	5.99E-28	****
Vldlr	0.5212	2.09E-04	***
Tgfbr3	0.5182	1.74E-03	**
Cxcl12	0.5669	4.11E-34	****
Slit3	0.5592	5.11E-27	****
Cdh11	0.6238	6.17E-114	****
Ephb4	0.5659	3.59E-33	****
Ephb2	0.5345	6.11E-10	****
Igf1r	0.6257	2.36E-117	****
Igf2r	0.6039	1.56E-80	****

Genes shown in Figure 2F

**Symbol**	**AUC**	**Multiple comparisons (FDR)**	
Hif1a	0.6919	8.46E-272	****
Sox11	0.6614	4.62E-193	****
Hes1	0.6346	1.76E-134	****
Mapk1	0.6237	1.25E-113	****
Ednra	0.5862	6.86E-56	****
Bmpr1a	0.5847	6.14E-54	****
Zeb2	0.5829	8.50E-52	****
Mapk3	0.5805	7.22E-49	****
Snai2	0.5740	1.60E-41	****
Bmp4	0.5707	5.38E-38	****
Tgfb2	0.5660	2.76E-33	****
Hand2	0.5652	1.82E-32	****
Cited2	0.5621	1.45E-29	****
Smo	0.5618	2.47E-29	****
Msx1	0.5578	9.15E-26	****
Aldh1a2	0.5349	4.05E-10	****
Mef2c	0.5348	4.41E-10	****
Hey1	0.5345	6.73E-10	****
Lef1	0.5333	2.54E-09	****
Jag1	0.5327	5.24E-09	****
Frzb	0.5312	2.60E-08	****
Kitl	0.5233	4.16E-05	****
Heyl	0.5225	8.26E-05	****
Acvr1	0.5202	4.55E-04	***
Notch1	0.5188	1.13E-03	**

Nes	0.5363	7.35E-11	****
Itga1	0.5212	2.11E-04	***
Mcam	0.5247	1.39E-05	****

Loxl3	0.5188	1.13E-03	**
Tgfbr3	0.5182	1.74E-03	**

Genes shown in Figure S4B.

Symbol	AUC	Multiple comparisons (FDR)	
Bmp4	0.5707	5.38E-38	****
Bmp1	0.5631	1.69E-30	****
Bmp7	0.5245	1.60E-05	****
Bmp2k	0.5184	1.53E-03	**
Bmpr1a	0.5847	6.14E-54	****
Bmpr2	0.5779	8.12E-46	****
Tgfb1i1	0.5596	2.28E-27	****
Tgfb1	0.5322	8.85E-09	****
Tgfb2	0.5660	2.76E-33	****
Tgfbi	0.5521	3.67E-21	****
Tgfbr1	0.5702	1.71E-37	****
Tgfbr3	0.5182	1.74E-03	****
Smad1	0.5400	6.18E-13	****
Smad2	0.5995	4.69E-74	****
Smad3	0.5214	1.85E-04	****
Smad4	0.5536	2.32E-22	****
Smad5	0.5859	1.88E-55	****
Smad6	0.5544	5.79E-23	****
Smad7	0.5577	1.05E-25	****
Id1	0.5381	7.73E-12	****
Id2	0.5992	1.49E-73	****
Id3	0.6577	2.28E-184	****
Id4	0.4989	6.04E-01	ns
Bmper	0.5084	2.00E-01	ns
Grem1	0.4955	8.09E-01	ns
Nog	0.4930	9.10E-01	ns

Genes shown in Figure S4C.

Symbol	AUC	Multiple comparisons (FDR)	
Wnt2	0.5622	1.04E-29	****
Wnt4	0.5373	1.97E-11	****
Wnt5a	0.5224	8.62E-05	****
Wnt5b	0.5170	3.63E-03	**
Wnt11	0.5382	6.85E-12	****
Lrp1	0.6014	8.40E-77	****
Lrp4	0.5128	3.40E-02	*
Lrp5	0.5190	1.03E-03	**
Lrp6	0.5667	5.68E-34	****
Fzd1	0.5569	5.49E-25	****
Fzd2	0.6285	1.50E-122	****
Fzd3	0.5716	6.09E-39	****
Fzd4	0.5156	8.07E-03	**
Fzd7	0.5438	2.61E-15	****
Axin1	0.5149	1.22E-02	*
Axin2	0.5380	8.93E-12	****
Gsk3a	0.5639	2.88E-31	****
Gsk3b	0.6794	8.34E-238	****
Ctnnb1	0.7234	0.00E+00	****
Tcf7	0.5259	4.86E-06	****
Tcf7l1	0.5400	5.88E-13	****
Tcf7l2	0.6970	1.86E-286	****
Lef1	0.5333	2.54E-09	****

Genes shown in Figure S4D.

Symbol	AUC	Multiple comparisons (FDR)	
Notch1	0.5188	1.13E-03	**
Notch2	0.5410	1.50E-13	****
Notch3	0.5466	3.72E-17	****
Jag1	0.5327	5.24E-09	****
Rbpj	0.6420	2.30E-149	****
Maml1	0.5363	7.58E-11	****
Maml3	0.5373	1.99E-11	****
Hes1	0.6346	1.76E-134	****

Genes shown in Figure 3L.

Symbol	AUC	Multiple comparisons (FDR)	
Igfbp4	0.7461	0.00E+00	****
Col3a1	0.6793	5.25E-238	****
Fbln1	0.6766	2.45E-230	****
Mfap4	0.6567	3.24E-182	****
Sfrp2	0.6120	2.04E-93	****
Ptn	0.6017	2.06E-77	****
Col18a1	0.6007	9.57E-76	****
Bmp4	0.5707	5.38E-38	****

					Postn	0.5522	2.78E-21	****
					Mmp23	0.5470	1.86E-17	****
					Spon1	0.5467	2.94E-17	****
					Col4a1	0.5422	2.91E-14	****
					Loxl1	0.5399	7.10E-13	****
					Angptl2	0.5260	4.20E-06	****
					Angpt2	0.5245	1.56E-05	****
					Col4a2	0.5231	5.07E-05	****
					Col16a1	0.5187	1.21E-03	**
					Hapln1	0.5186	1.30E-03	**
					Adamts18	0.5172	3.23E-03	**
					Gal	0.5158	7.17E-03	**
					Clec11a	0.5132	2.92E-02	*

NicheNet heatmap genes shown in Figure 2G-H in DP cells

Ligand	AUROC	AUPR	Corrected AUPR	Pearson
Bmp4	0.746	0.110	0.085	0.182
Tgfb1	0.730	0.098	0.074	0.175
Col4a1	0.757	0.085	0.061	0.162
Tgfb3	0.719	0.081	0.056	0.154
Copa	0.741	0.080	0.055	0.149
Wnt5a	0.704	0.077	0.053	0.143
Adam9	0.730	0.075	0.050	0.140
Col2a1	0.723	0.071	0.047	0.140
Dkk2	0.721	0.071	0.047	0.139
Edn3	0.713	0.079	0.055	0.138

Left: genes upregulated in CD31hi vs CD31lo WT IAHCs (Figure S6F). Right. Genes downregulated in the KO CD31hi vs WT CD31hi IAHCs.

Symbol	AUC	Multiple comparisons (FDR)			Symbol	AUC	Multiple comparisons (FDR)	
Pecam1	0.9756	1.96E-07	****		Pecam1	0.7497	1.10E-03	**
Cdh5	1.0000	7.99E-08	****		*Cdh5*	0.8028	4.27E-05	****
Kit	0.6375	4.07E-01	ns		*Kit*	0.7725	2.91E-04	***
Sox7	0.9268	8.86E-07	****		*Sox7*	0.6792	3.16E-02	*
Sox17	0.9634	1.79E-07	****		*Sox17*	0.7736	2.62E-04	***
Sox18	0.9850	8.49E-08	****		*Sox18*	0.8926	5.09E-08	****
Hif1α	0.6563	2.76E-01	ns		*Hif1a*	0.7927	8.21E-05	****
Bmpr2	0.8503	1.25E-04	***		*Bmpr2*	0.6235	2.12E-01	ns

Table S5. Summary of CFU-C data obtained from PDGFRβ$^{+/+}$ (WT), PDGFRβ$^{+/-}$ (HET) and PDGFRβ$^{-/-}$ (KO) MSC cell lines co-cultured with BM LSKs (upper table) and AGM CD31^{+}ckit^{+} IAHC cells (bottom table) and statistical analyses performed. Related to Figure 3.

				number of MSC lines				Average CFU-Cs								Multiple comparisons			
Tissue	Day	Cell number	CFU-type	WT	HET	KO	KO+BMP4	WT	HET	KO	KO+Bmp4	Statistical test	p-value		Post-hoc	WT vs HET	WT vs KO	KO vs KO+Bmp4	WT vs KO+BMP4
Adult BM LSK co-cultured with E11 AGM-MSCs	7	50000	BFU-E	5	6	5	4	2,90	4,13	0,59	2,07	Kruskal-Wallis	0,0720	ns	Dunn's	ns	ns	ns	ns
			CFU-G					3,75	5,67	1,15	3,50	1-way ANOVA	0,3536	ns	Tukey's	ns	ns	ns	ns
			CFU-M					15,63	12,46	3,90	10,07	1-way ANOVA	0,2711	ns	Tukey's	ns	ns	ns	ns
			CFU-GM					3,97	4,70	0,89	3,10	Kruskal-Wallis	0,0633	ns	Dunn's	ns	ns	ns	ns
			CFU-GEMM					6,10	4,32	1,49	3,09	Kruskal-Wallis	0,0957	ns	Dunn's	ns	ns	ns	ns
			Total					32,37	31,28	8,01	22,06	Kruskal-Wallis	0,0274	*	Dunn's	ns	*	ns	ns

				number of MSC lines			Average CFU-Cs							Multiple comparisons		
Tissue	Day	Cell number	CFU-type	WT	KO	KO+BMP4	WT	KO	KO+Bmp4	Statistical test	p-value		Post-hoc	WT vs KO	KO vs KO+Bmp4	WT vs KO+BMP4
E11 WT AGM HSPCs (CD31+ckit+) co-cultured with E11 AGM-MSCs	7	50000	BFU-E	3	3	3	1,4	0	2,7	1-way ANOVA	0,0064	**	Tukey's	ns	ns	**
			CFU-G				3,0	2,2	6,0	1-way ANOVA	0,0798	ns	Tukey's	ns	ns	ns
			CFU-M				9,7	5,6	18,3	1-way ANOVA	0,0254	*	Tukey's	ns	ns	*
			CFU-GM				0,9	1,1	3,1	1-way ANOVA	0,0167	*	Tukey's	ns	*	*
			CFU-GEMM				0,8	0	1,7	1-way ANOVA	0,0002	***	Tukey's	**	**	***
			Total				15,7	8,9	31,8	1-way ANOVA	0,0069	**	Tukey's	ns	*	**

Table S6.

a. Summary of CFU-C data obtained from PDGFRβ^{+} and PDGFRβ^{-} cells sorted from E10 and E11 WT AGMs and statistical analyses performed. Related to Figure 5.

Tissue	CFU-C type	PDGFRβ-	PDGFRβ+	Statistical test	p value	Significance
E10 AGM	BFU-E	3.56	0.00	Unpaired t-test with Welch's correction	<0,0001	****
	CFU-G	3.56	0.00	Unpaired t-test with Welch's correction	<0,0001	****
	CFU-M	12.13	0.00	Unpaired t-test with Welch's correction	<0,0001	****
	CFU-GM	2.31	0.00	Unpaired t-test with Welch's correction	0.0001	***
	CFU-GEMM	5.38	0.00	Unpaired t-test with Welch's correction	0.0002	***
	Total	26.94	0.00	Unpaired t-test with Welch's correction	<0,0001	****
E11 AGM	BFU-E	3.42	0.00	Unpaired t-test with Welch's correction	0.0003	***
	CFU-G	4.25	0.00	Unpaired t-test with Welch's correction	<0,0001	****
	CFU-M	25.08	0.00	Unpaired t-test with Welch's correction	0.0002	***
	CFU-GM	3.50	0.00	Unpaired t-test with Welch's correction	0.0026	**
	CFU-GEMM	5.58	0.00	Unpaired t-test with Welch's correction	0.0087	**
	Total	41.83	0.00	Unpaired t-test with Welch's correction	0.0002	***

b. Summary of CFU-C data obtained from TdTomato+ and TdTomato- cells sorted from E10 and E11 PDGFRβ-Cre;tdTomato AGMs and statistical analyses performed. Related to Figure 5.

Tissue	CFU-C type	Tomato-	Tomato+	Statistical test	p value	Significance
E10 AGM	BFU-E	4.00	0.00	Mann-Whitney U test	0.0003	***
	CFU-G	3.83	0.08	Mann-Whitney U test	0.0002	***
	CFU-M	14.33	1.08	Unpaired t-test with Welch's correction	0.0001	***
	CFU-GM	2.75	0.00	Mann-Whitney U test	0.0002	***
	CFU-GEMM	4.25	0.25	Mann-Whitney U test	0.0003	***
	Total	29.17	1.42	Mann-Whitney U test	0.0002	***
E11 AGM	BFU-E	3.50	1.63	Unpaired t-test with Welch's correction	0.0837	ns
	CFU-G	3.50	1.13	Unpaired t-test with Welch's correction	0.0425	*
	CFU-M	35.38	10.25	Unpaired t-test with Welch's correction	<0,0001	****
	CFU-GM	2.63	1.38	Unpaired t-test with Welch's correction	0.0602	ns
	CFU-GEMM	3.13	1.75	Unpaired t-test with Welch's correction	0.3204	ns
	Total	48.13	16.13	Unpaired t-test with Welch's correction	0.0004	***

Table S7. Summary of CFU-C data obtained from PDGFRβ+/- cells sorted from C57Bl6/j (WT) E14 FL and adult BM (upper table) and TdTomato+/- cells sorted from PDGFRβ-Cre;tdTomato E14 FL and adult BM, and statistical analyses performed (bottom table). Related to the Figure 7.

Tissue	Experiment	Cell sorted	Cells plated*	BFU-E	CFU-G	CFU-M	CFU-GM	CFU-GMEM	Total
E14 FL	Sort 1	PDGFRβ-	200000	13	15	42	24	41	135
		PDGFRβ+	1204	0	0	0	0	0	0
	Sort 2	PDGFRβ-	200000	15	11	39	24	38	127
		PDGFRβ+	1211	0	0	0	0	0	0
	Sort 3	PDGFRβ-	200000	23	11	33	24	37	128
		PDGFRβ+	1212	0	0	0	0	0	0
Adult BM	Sort 1	PDGFRβ-	200000	8	9	26	16	25	84
		PDGFRβ+	1195	0	0	0	0	0	0
	Sort 2	PDGFRβ-	200000	10	11	24	18	29	92
		PDGFRβ+	942	0	0	0	0	0	0
	Sort 3	PDGFRβ-	200000	11	9	20	19	21	80
		PDGFRβ+	1208	0	0	0	0	0	0
	Sort 4	PDGFRβ-	200000	9	9	23	17	28	86
		PDGFRβ+	671	0	0	0	0	0	0
	Sort 5	PDGFRβ-	200000	8	9	27	18	24	86
		PDGFRβ+	721	0	0	0	0	0	0

*****all PDGFRβ^{+} cells sorted correspond to one embryo equivalent (ee)

Tissue	CFU-C type	Tomato-	Tomato+	Statistical test	p value	Significance
E14 FL	BFU-E	1153.54	299.69	Unpaired t-test with Welch's correction	0.0314	*
	CFU-G	778.64	222.20	Unpaired t-test with Welch's correction	0.0278	*
	CFU-M	2739.65	717.16	Unpaired t-test with Welch's correction	0.0183	*
	CFU-GM	1557.27	397.48	Mann-Whitney U test	0.1000	ns
	CFU-GEMM	2912.68	635.92	Unpaired t-test with Welch's correction	0.0242	*
	Total	9141.77	2272.45	Unpaired t-test with Welch's correction	0.0082	**
adBM	BFU-E	4487.78	430.64	Unpaired t-test with Welch's correction	0.0540	ns
	CFU-G	6282.89	592.13	Unpaired t-test with Welch's correction	0.0106	*
	CFU-M	20344.60	1211.18	Mann-Whitney U test	0.0286	*
	CFU-GM	6881.26	565.22	Unpaired t-test with Welch's correction	0.0241	*
	CFU-GEMM	8377.19	619.05	Unpaired t-test with Welch's correction	0.0091	**
	Total	46373.72	3418.21	Unpaired t-test with Welch's correction	0.0067	**
